# Site-specific opening of the blood-brain barrier by extracellular histones

**DOI:** 10.1186/s12974-020-01950-x

**Published:** 2020-09-22

**Authors:** Nuria Villalba, Sheon Baby, Byeong J. Cha, Sarah Y. Yuan

**Affiliations:** 1 Department of Molecular Pharmacology & Physiology, University of South Florida, Morsani College of Medicine, Tampa, FL USA; 2 Department of Surgery, Morsani College of Medicine, University of South Florida, Tampa, FL USA

**Keywords:** Blood-brain barrier, Histones, Endothelial permeability

## Abstract

**Background:**

Increased extracellular histones in the bloodstream are known as a biomarker for vascular dysfunction associated with severe trauma or sepsis. There is limited information regarding the pathogenic role of circulating histones in neuroinflammation and cerebrovascular endothelial injury. Particularly, it remains unclear whether histones affect the blood-brain barrier (BBB) permeability function.

**Methods:**

The direct effects of unfractionated histones on endothelial barrier properties were first assessed in brain microvascular endothelial cell monolayers by measuring transendothelial electrical resistance and solute flux. This was followed by in vivo mouse experiments, where BBB function was assessed by quantifying brain tissue accumulation of intravenously injected tracers of different molecular sizes, and comparison was made in mice receiving a sublethal dose of histones versus sterile saline. In parallel, the endothelial barrier ultrastructure was examined in histone- and saline-injected animals under transmission electron microscopy, corresponding to the expression of tight junction and adherens junction proteins.

**Results:**

Histones increased paracellular permeability to sodium fluorescein and reduced barrier resistance at 100 μg/mL; these responses were accompanied by discontinuous staining of the tight junction proteins claudin-5 and zona ocludens-1. Interestingly, the effects of histones did not seem to result from cytotoxicity, as evidenced by negative propidium iodide staining. In vivo, histones increased the paracellular permeability of the BBB to small tracers of < 1-kDa, whereas tracers larger than 3-kDa remained impermeable across brain microvessels. Further analysis of different brain regions showed that histone-induced tracer leakage and loss of tight junction protein expression mainly occurred in the hippocampus, but not in the cerebral cortex. Consistently, opening of tight junctions was found in hippocampal capillaries from histone-injected animals. Protein expression levels of GFAP and iBA1 remained unchanged in histone-injected mice indicating that histones did not affect reactive gliosis. Moreover, cell membrane surface charge alterations are involved in histone-induced barrier dysfunction and tight junction disruption.

**Conclusions:**

Extracellular histones cause a reversible, region-specific increase in BBB permeability to small molecules by disrupting tight junctions in the hippocampus. We suggest that circulating histones may contribute to cerebrovascular injury or brain dysfunction by altering BBB structure and function.

## Background

Histones are small, positively charged nuclear proteins that strongly interact with negatively charged DNA forming complexes called nucleosomes [1]. Only recently has histones emerged as potent proinflammatory molecules with important extracellular locations and actions [2–5]. Increasing evidence supports the linkage between high levels of extracellular histones in the bloodstream with a variety of pathological conditions, including trauma, septic shock [5–7], and autoimmune diseases [8]. Histones also play important roles in sterile inflammation caused by hemorrhagic shock, ischemia-reperfusion injury, or chemically induced liver injury [9–11]. Histones, released from dying cells or as part of neutrophil-extracellular traps (NETs), have been associated with endothelial dysfunction, vascular leakage, coagulopathy, and cardiomyopathy [4, 5, 12, 13]. The association between high levels of histones and poor clinical outcome has also been reported. Circulating plasma histone protein levels in healthy individuals are ~ 2 μg/mL increasing up to ~ 200 μg/mL within 4 h of severe trauma [5]. Some studies suggest that histones act as ligands for toll-like receptors (TLRs), thus exerting their effect through activation of the innate immune system [10, 14].

The blood-brain barrier (BBB) is a specialized system composed of brain microvascular endothelial cells, which protects the brain from harmful substances in the bloodstream while supplying the brain with vital nutrients to maintain proper function [15, 16]. Unlike peripheral capillaries where the endothelium is discontinuous or loosely formed allowing relatively free exchange of substances, the cerebral endothelium is continuous with tightly sealed endothelial cells connected by cell–cell junction complexes. The polarized distribution of intercellular tight junction (TJ) proteins including zona-occludens-1 (ZO-1) and claudin-5 on the apical side of adherens junctions (AJs) is also remarkable on the brain microvascular endothelium compared to that in other organs [17, 18]. These unique structures make the BBB highly restrictive, especially to paracellular passage of molecules. In order to freely diffuse across the BBB, a molecule must fit the dual criteria of small molecular weight (under a 400–500 Da threshold) and high lipid solubility (equivalent to low hydrogen bonding) [19–21]. High molecular weight, water-soluble molecules do not favor crossing by transmembrane diffusion compared to low molecular weight molecules with high lipid solubility. Due to these characteristics, only few molecules are good candidates for BBB permeation.

The vascular endothelium is highly heterogenous and shows remarkably different sensitivities to circulating factors and exogenous activators across organs [22, 23]. The impact of histones on visceral organs may differ from that to the central nervous system (CNS) and particularly, the brain vasculature. To date, despite a vast majority of literature demonstrating histone-induced immune response and tissue damage in the lung, liver, kidney, and heart, whether and how extracellular histone proteins affect specialized endothelial barrier in the brain remains to be elucidated.

The present study hypothesizes that extracellular histones at high levels such as those seen in critical illness impair the BBB structure and function. Using a combination of in vitro, ex vivo, and in vivo approaches, we examined the direct effects of histones on brain endothelial barrier function and brain regional BBB integrity during systemic administration of histones. Results from this study provide novel insights into the molecular mechanisms of neuroinflammation.

## Methods

### Endothelial cell culture

Primary cerebral microvascular endothelial cell cultures were prepared from female and male C57bl/6 mouse brains (Cell Biologics, Chicago, IL; catalog no. C57-6023) and grown in the recommended culture medium (Cell Biologics, catalog no. M1168), split at a ratio of 1:2, and used at passage 2. Experimental series were designed using different batches of cells in order to provide biological replicates.

### Assessment of barrier function by electric cell-substrate impedance sensing assay

Mouse brain endothelial cell barrier function was determined by measuring the cell-cell adhesion barrier resistance to electric current using an electric cell-substrate impedance sensor (ECIS) system (Model ZƟ; Applied BioPhysics Inc., Troy, NY) as previously described [24]. Briefly, 250 μL of cell suspension (5 × 10^5^ cells per mL) were seeded to each well of two 8-well-ECIS cultureware arrays (8W1E+) following the manufacturer’s instructions. Resistance was recorded in real time with the following settings: alternating current (1 V), frequency (4 kHz) at 7-s intervals. Data were normalized to baseline measurements just prior to the onset of treatment (*t* = 0) and readings were acquired continuously for 24 h. Both data acquisition and processing were performed using the ECIS data analysis software supplied by Applied Biophysics. Treatment with ZVAD-fmk (100 μM), a cell permeable broad caspase inhibitor (Tocris Bioscience; catalog no. 2163/1), oleylamine (250 μM; Sigma-Millipore; catalog no. 909831) and cholesterol sulfate (250 μM; Cayman; catalog no.15106) was performed for 1 h prior to adding vehicle or histones. The data were represented as normalized resistance, which is the resistance measured after treatment over the resistance acquired before treatment introduction.

Since every cell type has its characteristic adhesion and growth, the ability of mouse brain endothelial cells to form a tight monolayer was assessed using ECIS. In order to confirm the formation of a mature BBB, cells were grown until a constant baseline resistance was obtained. Resistance values increased over time (growth phase) until they reached to stable transendothelial electrical resistance (TEER) values (plateau phase) indicating that cells formed matured cell-cell junctions approximately 5 days (∼ 125 h) later. Thus, cells were used for different in vitro experimental series (ECIS assays, transwell permeability, or immunofluorescence) 5 days after being plated. All TEER experiments were done in triplicate with at least 3 independent biological replicates. Data show mean ± S.E.M.

### Transwell permeability assay

Endothelial monolayer permeability experiments were conducted using brain endothelial cells grown at a density of 2 × 10^5^ cells on 0.33 cm^2^ gelatin-coated inserts of 0.4 μm pore size (Corning Inc., Tewksbury, MA; catalog no. 3401). Endothelial cells were cultured on the inserts for 5 days to allow the cells to form a monolayer and mature TJs (as previously assessed by TEER measurements, see below). On the day of the experiment, vehicle (saline) or histones (10, 25, 50, and 100 μg/mL) were added to the top chambers followed by NaFl or Texas Red 3-kDa dextran to a final concentration of 1 mg/mL. The diffuse flux of the tracers was measured by sampling media (100 μL) from the bottom chamber at 3 h after adding the molecular tracers and treatments. The concentration of each tracer diffusing from the top to the bottom chamber was determined using a SpectraMax M3 plate reader. The amount of dye was calculated by extrapolation from a standard curve of known NaFl (with excitation at 490 nm and emission at 520 nm) concentrations using linear regression. Apparent permeability coefficients (*P*_app_) of mouse brain endothelial monolayers were calculated as follows:

*P*_app_ = [*C*]/*t* × 1/*a* × *V*/[*L*], where [*C*] is the concentration of the tracer in the bottom chamber, *t* is time (in seconds), *a* is the cross-sectional area of the membrane (0.33 cm^2^), *V* is the volume of the bottom chamber (670 μL), and [*L*] is the initial tracer concentration in the side of the chamber loaded with the tracer (top chamber). Permeability coefficient was reported in units of cm s^–1^. Statistical analysis (Kruskal–Wallis and Dunnett’s post-hoc test) was performed using GraphPad Prism 8.0 software (San Diego, CA).

In a different set of experiments, the baseline flux of NaFl, 1-kDa, and Texas Red 3-kDa dextran was determined by taking aliquots from the bottom chamber at different time points and replacing them with equal amount of fresh warmed media. Flux was calculated as the slope of the best-fit line of accumulated tracer (μg/cm^2^) versus time (min), using the least-squares method. The amount of tracer accumulated in the bottom of the chamber increased linearly with time (steady-state phase). The extrapolation of the linear portion of the curve to the x-axis yielded the lag time (time required to reach steady-state).

### Immunocytochemistry

Brain endothelial cell monolayers plated on Lab-Tek® chamber slides (Thermofisher Scientific; catalog no. 1777399PK) were treated with vehicle or histones, washed in PBS containing 2 mM CaCl_2_ and 2 mM MgCl_2_, and fixed in 2% paraformaldehyde (PFA) in PBS for 20 min. Cells were rinsed in PBS, permeabilized for 10 min (in 0.1% Triton-X/PBS), and blocked (10% normal goat serum in 3% BSA/PBS) for 1 h at room temperature. Slides were incubated with primary antibodies ZO-1 (1:100; Thermofisher; catalog no. 40-2200) and claudin-5 (1:50; Thermofisher; catalog no. 35-2500) overnight at 4 °C, washed 7 times, and incubated with appropriate secondary Alexa Fluor® antibodies (diluted at 1:500). After washing, slides were then cover slipped using ProLong™ diamond antifade mountant with DAPI and confocal microscopy imaging was performed using a Leica DMi8 STED confocal microscope. Z-stack of images were acquired using a HC PLAPO 63×/1.4 NA objective. Images were reconstructed using Leica Application Suite X software (LAS X).

### In vitro histone cytotoxicity assay

Cytotoxicity was evaluated with propidium iodide (PI; Invitrogen; catalog no. P3566) as a cell viability indicator for dead (PI permeable) and viable (PI impermeable) cells. In brief, mouse brain endothelial cells were grown to 90% confluence in 6-well plates and treated with vehicle (saline) or histones (10, 25, 50, 100, and 250 μg/mL) in culture media. After 3 h of incubation, the medium was removed to a fresh tube, and the remaining cells were trypsinized and transferred to the same tube. The cells were pelleted and resuspended in 1 mL PBS containing 1 mg/mL PI. Events (5 × 10^4^) were acquired on a BD LSR II flow cytometer (BD Biosciences, San Jose, CA), and data were analyzed using FlowJo 7.6.4. software. Cell survival rates were normalized by vehicle-treated cells (designated as 100%).

### Ex vivo pressure myography

Following euthanasia, the brain was carefully removed and placed in ice-cold phosphate-buffered saline solution (PSS) containing (in mM): 119 NaCl, 4.7 KCl, 20 NaHCO_3_, 1.1 KH_2_PO_4_, 1.2 MgSO_4_, 1.6 CaCl_2_, and 10 glucose (pH 7.4). Posterior cerebral arteries isolated from saline- or histone-injected mice were carefully dissected out of surrounding tissue and cut into ~ 2 mm segments. Arterial segments were cannulated in an arteriograph chamber (Living Systems Instrumentation, St Albans, VT) and superfused with warm (37 °C) PSS. Before each experiment, arteries (internal diameter of ~ 100 μm when pressurized at 10 mm Hg) were allowed to equilibrate for a period of 10 min at 10 mmHg intravascular pressure and contractile responsiveness was assessed by exposure to isotonic hyperpolarizing solution (60 mM KCl-PSS made by equimolar substitution of KCl for NaCl in PSS). Following equilibration, intravascular pressure was slowly raised from 10 to 60 mmHg (in vivo pressure). Pressurized arteries were mounted on an inverted light microscope equipped with a CCD camera and edge-detection software for continuous monitoring of internal diameter (IonOptix, Milton, MA). Only cerebral arteries that exhibited myogenic tone were used. Maximal diameter was obtained at the end of each experiment by superfusing Ca^2+^-free PSS containing a calcium channel blocker (100 μM, diltiazem). Vascular (myogenic) tone was measured in pressurized vessels in vitro as the difference in vessel diameter under active conditions (presence of calcium in PSS) vs. passive relaxed conditions (calcium-free PSS) obtained at the same intravascular pressure and calculated as a percentage of the Ca^2+^-free diameter to normalize for differences in diameter between arteries. Endothelial cell function was determined by measuring the dilatory response to activation of endothelial SK/IK channels with NS309 (Tocris Biosciences; catalog no. 3895) [25, 26].

### Animal procedures

All studies were performed in accordance with the University of South Florida Institutional Animal Care and Use Committee based on the National Institute of Health guidelines for care and usage of laboratory animals. Female and male C57bl/6 mice (12–16 weeks of age) were bred and housed in cages on a 12-h light cycle with ad libitum access to water and a standard laboratory diet. Mice were anesthetized with isoflurane (2.5%) and then administered a single intravenous injection, through the retro-orbital venous sinus, of histones at 45 mg/Kg (highest non-lethal dose of histones that corresponds to circulating histone levels of ~ 80 μg/mL [27]) or the same volume of sterile saline. Mixture of unfractionated histones (consisting in 7.5% H1, 20.8% H2A, 32.5% H2B, 10.2% H3, and 28.9% H4 as previously reported [26]) isolated from calf thymus was used in this study (dissolved in sterile saline at 10 mg/mL; molecular weights of histones are 11- to 21-kDa depending on the fraction; Roche® distributed by Millipore-Sigma; catalog no. 10223565001). Mice were euthanized at 24 h, 3, 7, or 14 days after histone or saline injection depending on the experimental series as indicated in the results and figure legends.

### Measurement of BBB permeability in vivo

The paracellular permeability of BBB to different molecular size tracers was determined by ratiometric assays of amount of dye/mg of tissue. Two groups of mice (saline or histones) were intravenously injected through the retro-orbital venous sinus with sodium fluorescein (NaFl; 5 μL/g of a 100 mg/mL NaFl solution; 376 Da; Stokes radius 0.45 nm; Sigma-Millipore; catalog no. 6377), Alexa Fluor 555–cadaverine (5 μg/g of a 1 mg/mL solution; 1-kDa; Invitrogen; catalog no. A30677), Alexa Fluor 648–3-kDa dextran (5 μg/g of a 1 mg/mL solution; Invitrogen; catalog no. D34681), and 1-kDa infrared dye (IRdye; 5 μg/g of a 1 mg/mL solution; Sigma-Millipore; catalog no. SCJ4600056) at 24 h post-saline or histones injection. Tracers were allowed to distribute in vivo for 30 min (NaFl) or 1 h (Alexa Fluor 555–cadaverine, 3-kDa dextran and 1-kDa cadaverine) in freely moving mice. Then, mice were anesthetized with urethane (1.75 mg/Kg; intraperitoneally) and transcardially perfused with ice-cold 0.1 M phosphate buffer (PBS; pH 7.4). After dissection, brains were harvested, weighed, and homogenized with 20% trichloroacetic acid and then diluted with equal volumes of borate buffer 0.05 M (for NaFl) or 1% Triton X-100 in PBS (for 1-kDa cadaverine and 3-kDa dextran). Tissue homogenates were centrifuged at 12,000×*g* for 20 min at 4 °C and proteins were precipitated with ethanol. The fluorescence of a 100-μL aliquot of the brain samples along with 100 μL of blank or standards was measured on a fluorescence microplate reader (SpectraMax M3; Molecular Devices, Sunnyvale, CA). The concentrations of the samples were within the linear range of the standard curve. Amount of dye (ng) was normalized per brain weight (mg).

The site-specific disruption of the BBB was determined in a subset of brains isolated from animals injected with 1-kDa IRdye. Ventral and dorsal brains were scanned by an ex vivo near-infrared (NIR) imaging system (Odyssey CLx; Li-Cor, Lincoln, NE) using 700- and 800-nm excitation. Brains were excised and sectioned into 1 mm slices from rostral to caudal using a coronal Adult Mouse Brain Slicer Matrix (Leica). Subregional analysis of tracer distribution was measured in coronal sections by measuring mean fluorescence intensity of nine coronal sections covering + 3 to − 5 mm from anterior to posterior relative to bregma. Mean fluorescence intensity for each coronal section was plotted against the distance to bregma (mm) to evaluate the progression of the leakage throughout the brain.

### Immunoblotting analysis

Cortex and hippocampus from saline- and histone-injected mice were macroscopically dissected, and all visible white matter was discarded. Tissue was homogenized in 1 mL RIPA lysis buffer (Millipore; catalog no. 20-188) containing protease and phosphatase inhibitors (Roche; catalog no. 11697498001 and 04906845001). The supernatant of a 12,000×*g* spin was used for immunoblotting. Homogenates containing 20 μg of protein (Pierce™ BCA kit) were run on a 4–20% Tris-Glycine gel and transferred onto a nitrocellulose membrane (BioRad). The membrane was blocked in Li-Cor blocking buffer for 1 h at room temperature, probed with primary antibodies against occludin (1:500; BD Biosciences, catalog no. 611091), ZO-1 (1:500; Thermofisher; catalog no. 40-2200), VE-cadherin (1:1000; Abcam; catalog no. ab205336), GFAP (1:1000; Sigma-Millipore; Catalog no. MAB360), iBA1 (1:500; Abcam; catalog no. ab178846), and beta-actin (1:1000; Li-Cor; catalog no. 926-42212 and 926-42210). Membranes were washed with PBS-T (0.1% Tween 20) and probed with Li-Cor IRDye secondary antibodies in blocking buffer containing 0.1% Tween-20 + 0.05% SDS for 1 h at room temperature. The washed membranes were then imaged on a Li-Cor Odyssey CLx imaging system and blots were analyzed using the Image Studio Lite 5.2.5 software. Relative protein abundance was obtained by densitometry analysis. The signal intensity of all protein bands was normalized to the beta-actin loading control band (relative abundance).

### Transmission electron microscopy

Mice were anesthetized at 24 h after saline- or histone injection and fixed by transcardial perfusion at an intravascular pressure of 60 mmHg with a solution containing 2% glutaraldehyde in 0.1 M sodium cacodylate buffer, pH 7.4 (Electron Microscopy Sciences; catalog no. 16536-15). Tissue samples from the relevant areas of the brain (cerebral cortex and hippocampus) were dissected from intact brains and post-fixed in 2% glutaraldehyde in 0.1 M sodium cacodylate for an additional 24 h at 4 °C. Samples were rinsed in 0.1 M sodium cacodylate (Electron microscopy sciences; catalog no. 11652) for 6 h and subsequently immersed in 2% aqueous osmium tetroxide with 1.5% potassium ferrocyanide buffer for 4 h at room temperature. After 3 rinses in 0.1 M cacodylate buffer for a total time of 15 min, dehydration steps followed using a series of graded ethanol (35, 50, 70, 95, and 100%) and a final change into acetone prior to resin infiltration (Embed810 eponate). Infiltration steps included immersion of samples in intermediate solutions of 1:1 and 2:1 resin to acetone for 1 h each, prior to final immersion in pure fresh resin overnight followed by embedding in silicon flat molds and polymerization in a 60 °C oven. Ultrathin (90–100 nm) sections were obtained using an ultracut microtome without additional contrast enhancement. A series of non-overlapping images capturing the entire capillary circumference were obtained with a JEOL1400 transmission electron microscope equipped with a side mounted Gatan Orius digital camera.

### Tissue processing and Immunohistochemistry

Anesthetized mice were transcardially perfused through the left ventricle with ice-cold PBS. Mouse brains were cut through the hippocampus (~ 1.46 mm caudal to bregma), embedded in Tissue-Tek® O.C.T. compound (Sakura Finetek USA Inc., Torrance, CA), and snap-frozen (“fresh frozen tissue”). Coronal sections were sectioned at 20 μm on a Leica CM1950 cryostat and stored at − 80 °C until use. Cryosections were thawed and immediately fixed in either ice-cold methanol (claudin-5, ZO-1) or 2% PFA (VE-cadherin) for 15 min.

For glial fibrillary acidic protein (GFAP) and ionized-calcium binding adapter molecule-1 (iBa1) immunostaining, animals were transcardially perfused via the left ventricle with 4% PFA in PBS. After 1 day of post-fixation in the same fixative, brains were embedded in paraffin and then sectioned (4 μm) using a microtome.

Mouse brain sections were permeabilized and blocked for 1 h at room temperature in blocking buffer (0.3% Triton X-100 and 10% normal goat serum in 3% BSA/PBS) and incubated overnight at 4 °C with the following primary antibodies: GFAP (1:500; Genetex; catalog no. GTX108711), iBA1 (1:250; Abcam; catalog no. ab178847), claudin-5 (1:50; Thermofisher; catalog no. 35-2500), VE-cadherin/CD144 (1:100; BD Pharmingen; catalog no. 555289), and ZO-1 (1:100; Thermofisher; catalog no. 40-2200) diluted in blocking buffer at constant agitation. To visualize brain microvessels, sections were incubated with Dylight 488-conjugated *Lycopersicum esculentum* lectin (1:100; Vector laboratories, Burlingame, CA; catalog no. DL-1174) during the primary antibody incubation. After the washing steps, sections were incubated with fluorescent secondary antibodies (1:500; Alexa Fluor® dyes; Invitrogen) diluted in blocking buffer for 1 h at room temperature. Coronal brain sections were subsequently washed for 1 h with washing buffer, mounted in ProLong™ diamond antifade mountant with DAPI (Life Technologies; catalog no. P36966) and imaged using a Leica DMi8 STED confocal microscope using a 20× HC PLAPO, N.A. 0.75 objective (Leica Microsystems, Rueil-Malmaison, France). Images were acquired as z-stack projections and pseudo-coloring was performed to better visualize brain microvessels using Leica Application Suite X software (LAS X). Images of the confocal microscopy observations (512 by 512 pixels) were acquired sequentially, between stacks, to eliminate spectral crosstalk from the different channels. For each brain slice, one random field per animal was selected from the cortex (40x magnification for TJ and AJ proteins) and hippocampus (× 20 magnification). Six to nine separate vessels from each image were analyzed, and the data averaged and expressed as percentage. The area of ZO-1, claudin-5, or VE-cadherin-positive staining was normalized to the total area of lectin-positive microvessels using the Fiji hand free drawing tool to trace the outline of individual microvessels and the FIJI area measurement tool. The *n* value represents the number of animals in the group, with at least 5–6 mice analyzed. Overlays and maximum-intensity projections of 20 single slices from z-stack images were reconstructed post-acquisition by using ImageJ package FIJI, version 2.0 (fiji.sc/Fiji) (National Institutes of Health, Bethesda, MD) to obtain the presented images. Optimal antibody concentrations were first assessed by titration to determine working concentrations used for experiments. Gain, digital offset, and laser intensity were kept standardized.

### Brain water content

The brain water content was determined from differences in the wet and dry weight of the whole brain as follows: [(wet weight − dry weight)/wet weight] ×100%.

### Alexa Fluor 594-labeled histones

Histones were conjugated with a fluorescent NHS ester dye following the manufacturer’s instructions. Briefly, 10 mM stock solution of Alexa Fluor 594–NHS succinimidyl ester (Invitrogen; catalog no. A20009) prepared in anhydrous DMSO was added to a 10 mg/mL solution of calf thymus histones prepared in 50 mM borate buffer (pH 9.0) while gently stirring at room temperature. The reaction was quenched by adding 100 μL of 50 mM Tris-glycine after 1 h. The protein-dye conjugate was purified to remove excess of unbound dye using centrifugal Amicon® ultra-15 filters with a 3-kDa molecular weight cutoff (Sigma-Millipore; catalog no. UFC900308). The protein concentration was measured using a Pierce™ BCA protein assay kit (Pierce/Thermo Fisher; catalog no. 23227). Finally, the histone-dye conjugate was aliquoted and stored at − 20 °C.

### Statistical analysis

Data are presented as means ± standard error of the mean (S.E.M.). For animal experiments, female and male mice were randomly selected and used as biological replicates. For arteriography experiments, one artery per animal was used. Normality of the data was tested using the Shapiro–Wilk test for normality. For data that were not normally distributed (nonparametric data), the Mann–Whitney test (two groups, one variable) or Kruskal–Wallis test followed by Dunn’s correction (> 2 variables) was used. Data with normal distribution were analyzed by a two-tailed unpaired Student’s *t*-test (one variable) or one-way ANOVA with Dunnett’s or Tukey’s correction (> 2 variables). When two factors were analyzed, data were analyzed using two-way ANOVA with Tukey’s correction. Statistical test used to analyze the data is described in the figure legends. In all tests, a 95% confidence interval was used, for which *P* < 0.05 was considered a significant difference. Statistical analyses and graphs were performed in Prism 7.0 software (GraphPad, La Jolla, CA).

## Results

### High levels of histones attenuate barrier integrity and TJ staining in brain microvascular endothelial cell monolayers

High electrical resistance is a defining characteristic of the BBB and is commonly used to evaluate endothelial barrier integrity (“endothelial tightness”) [24, 28, 29]. Measurements of TEER and transwell permeability assays were used to assess functional endothelial barrier integrity alongside immunocytochemistry to test whether changes in endothelial permeability were accompanied by a reorganization of TJs. First, we used ECIS to monitor barrier formation of mouse brain cells (Supplemental Fig. 1). The tightness of the cell–cell contacts of primary mouse brain endothelial cells obtained as raw TEER readings was ∼ 2500 Ω (35–50 Ωcm^2^; Supplemental Fig. 1). Measurements showed that TEER values plateaued by day 5 indicating the formation of a tight endothelial barrier. High TEER values were maintained for another 4 days after the permeability barrier was achieved (Supplemental Fig. 1). NaFl and 1-KDa tracers were used to verify the impact of the molecular size on diffusion across brain endothelial cell monolayers. The accumulation of NaF and 1-KDa tracers increased with time showing higher flux and shorter lag time for NaF compared to the larger size 1-kDa tracer (Supplementary Fig. 2). However, Texas Red 3-kDa dextran did not permeate across the brain endothelial cell monolayer (data not shown). These results indicate that brain endothelial cells exhibit selective permeability to small molecular size tracers.

**Fig. 1:**
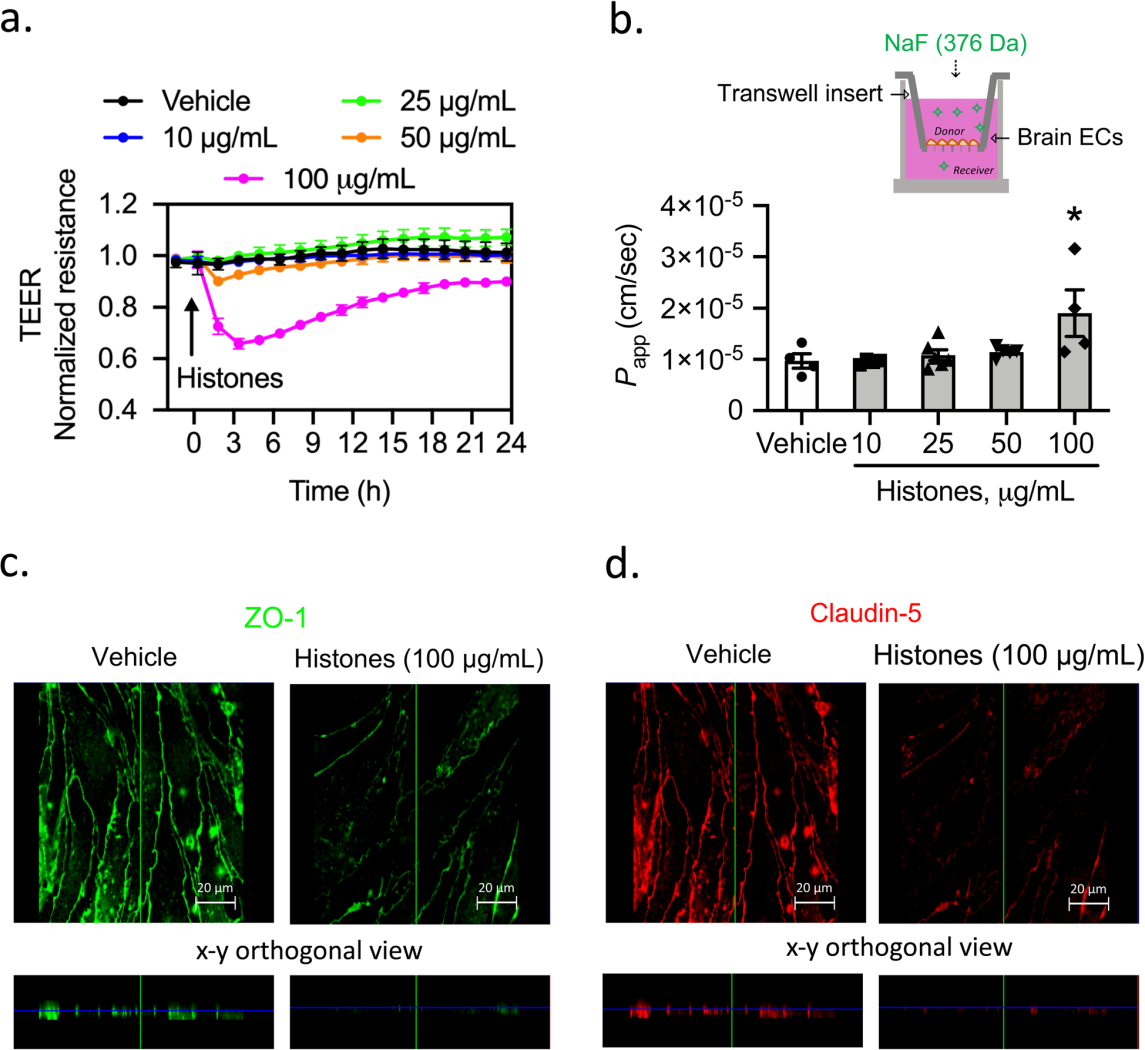
High levels of histones attenuate barrier integrity and TJ staining in mouse brain endothelial cell monolayers. **a** TEER measurements across confluent monolayers of brain endothelial cells treated with vehicle (saline) or histones (10, 25, 50, and 100 μg/mL). Mean ± S.E.M. Treatments were done in triplicate with four independent biological replicates. **b** The apparent permeability coefficient (*P*_app_) of brain endothelial cell monolayers to NaFl after 3 h of incubation with either vehicle or histones. Kruskal-Wallis test followed by Dunn’s test for multiple comparisons; *n* = 4–5 transwells per group; *P = 0.028. Schematic diagram of transwell permeability assay. **c**, **d** Immunocytochemical analysis of ZO-1 (**c**) and claudin-5 (**d**) in response to treatment of brain endothelial cells with vehicle (saline) or histones at 100 μg/mL. Images are representative of three independent biological replicates. Scale bar = 20 μm

Next, we evaluated whether histones affect the barrier integrity of endothelial cells by measuring TEER. Histones at a concentration of 100 μg/mL (~ 7 μM) induced a 35% reduction in TEER showing a peak TEER value at 3 h after the addition of histones (Fig. 1a). Furthermore, we used transwell permeability assays to assess the permeability apparent (*P*_app_) of the brain endothelial cell monolayers to NaFl following histone treatment. Only the highest concentration of histones (100 μg/mL) yielded a statistically significant increase in permeability of brain endothelial cells to NaFl (Fig. 1b).

Permeability data were reinforced by immunostaining of junctional proteins ZO-1 and claudin-5, which revealed a reorganization of TJs in cells exposed to histones. Cells treated with histones at 100 μg/mL showed discontinuous and more diffuse staining of ZO-1 and claudin-5 compared to the distinctly pattern localized at the cell borders in cells treated with vehicle (Fig. 1c, d).

### High levels of histones are not toxic to brain endothelial cells or native cerebrovascular endothelium

**Fig. 2:**
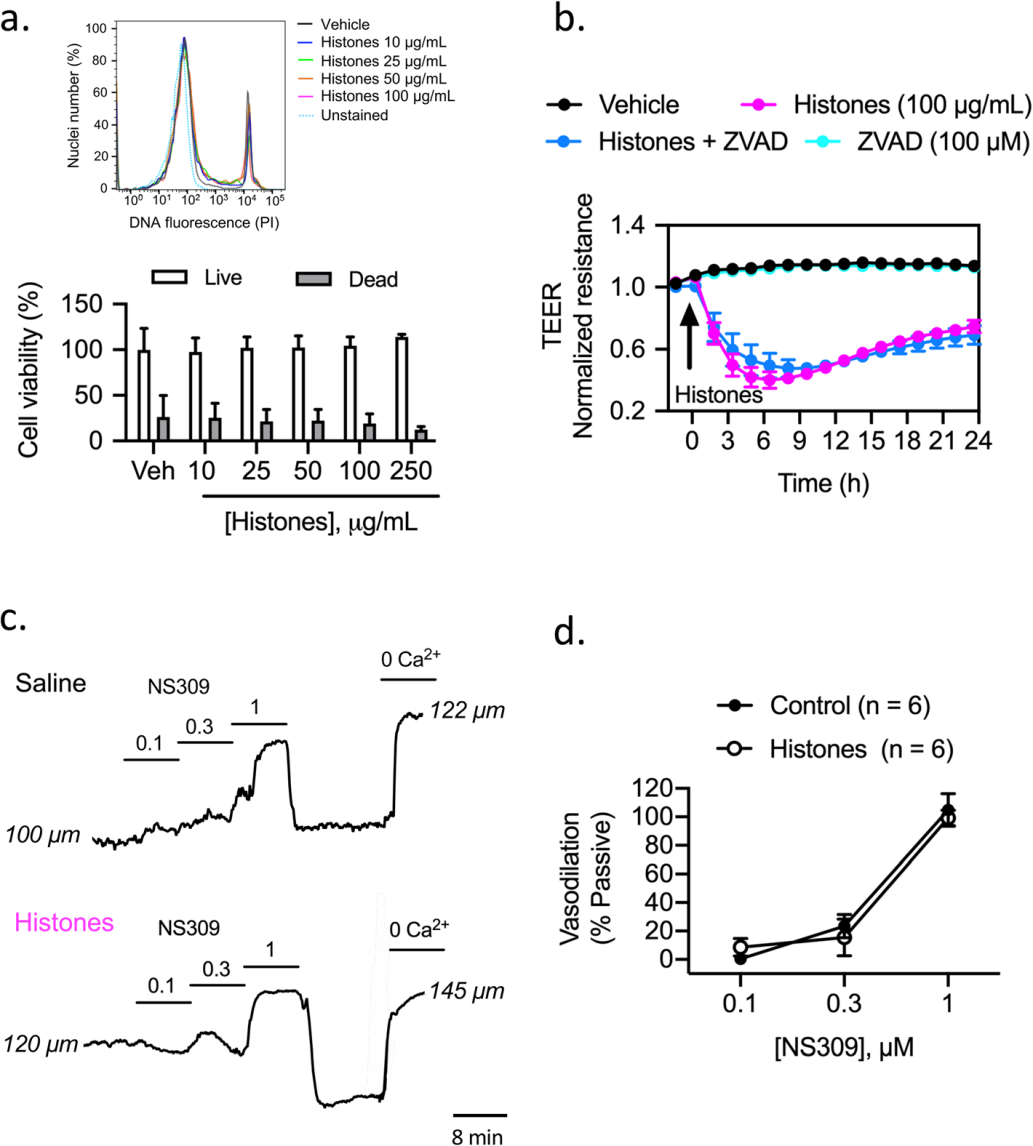
Extracellular histones are not cytotoxic to mouse brain endothelial cells or native cerebrovascular endothelium. **a** Cytotoxic effect of histones (10, 25, 50, 100, and 250 μg/mL) on brain endothelial cells assessed by PI uptake and analyzed by flow cytometry after 3 h treatment. (Top) Histograms showing percentage of total PI uptake in brain endothelial cells exposed to different histone concentrations as representative from one experiment. (Bottom) Summary data showing histone-induced cytotoxicity expressed as percentage cell viability relative to vehicle-treated cells after exposure of histones. Mean ± S.E.M. of 6–8 experiments. **b** TEER measurements of brain endothelial cell monolayers treated with vehicle (saline) or histones (10, 25, 50, and 100 μg/mL) in the presence of the pan caspase inhibitor ZVAD-fmk (100 μM; 1 h treatment). Mean ± S.E.M. Treatments were done in triplicate with three independent biological replicates. **c** Representative traces illustrating lumen diameter responses in a cannulated posterior cerebral artery pressurized to 60 mmHg isolated from a saline and a histone-injected mouse. Endothelial-dependent dilations were assessed by SK/IK channel activation using NS309 (0.1–1 μM). **d** Summary data showing percent dilation to NS309 relative to passive (0 calcium) in arteries isolated from saline- and histone-treated mice. Data are presented as means ± S.E.M

Previous studies have shown that calf thymus histones are toxic to culture cells including pulmonary endothelial cells [4, 5, 30], intestinal epithelial cells [30], smooth muscle cells [31, 32], and freshly isolated erythrocytes [33]. Next, we investigated whether histones induce cell death in brain endothelial cells. Cytotoxic effects of histones were evaluated by flow cytometry using PI. Histones (10, 25, 50, 100, and 250 μg/mL) added to brain endothelial cells for 3 h did not affect cell death compared to vehicle-treated cells (Fig. 2a). Moreover, histone-induced decrease in TEER was not inhibited by the pan caspase inhibitor ZVAD-fmk (100 μM), suggesting that the drop in TEER was not the result of caspase-dependent apoptosis (Fig. 2b). Additionally, the extent of endothelial cytotoxicity in vivo was also determined. The effect of circulating histones on the native cerebrovascular endothelium was studied on intact mouse pressurized-cerebral arteries isolated from saline- and histone-injected mice. Circulating histones did not alter endothelial reactivity of myogenically active mouse cerebral arteries as evidenced by intact endothelial-dependent dilations to the vasodilator NS309 (Fig. 2c, d). These data demonstrate that histones are not toxic to endothelial cells of the cerebral vasculature.

### Circulating extracellular histones induce a reversible and size-selective opening of the BBB

**Fig. 3:**
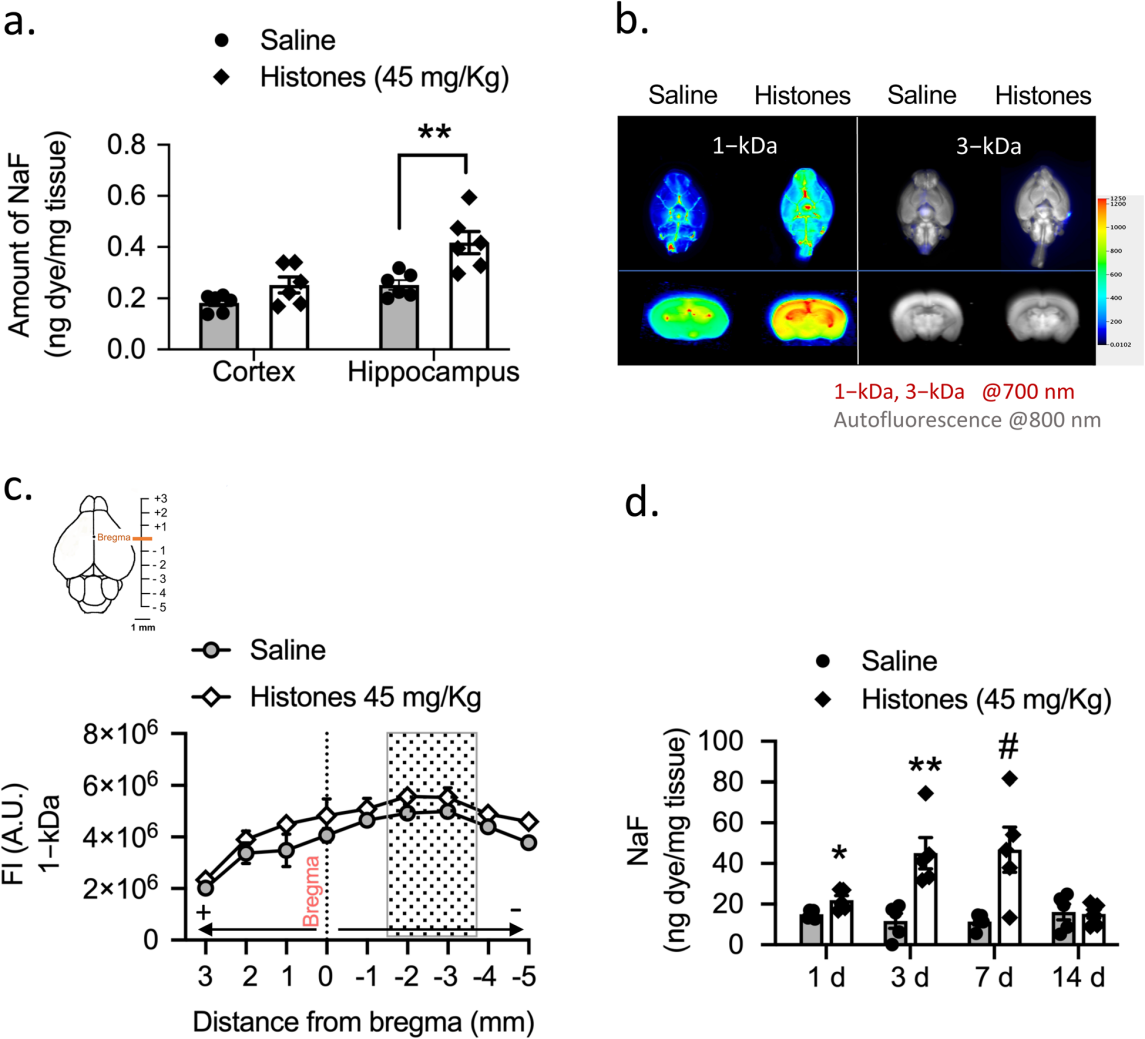
Circulating levels of extracellular histones increase BBB permeability. **a** Quantitative analysis showing NaFl uptake in cerebral cortex and hippocampus in saline- compared to histones-treated group. Mann–Whitney test; **P = 0.0043; Mean ± S.E.M.; *n* = 6 mice per group. **b** Representative NIR fluorescence images of the ventral whole-brains (upper) and coronal sections (bottom) across the hippocampus of saline- and histone-treated mice showing the distribution of 1-kDa and 3-kDa tracers within the brain. For images source data, see Supplementary Fig. 3a, b. **c** Quantification of whole-slide brain sections (+ 3 to − 5 mm from bregma) fluorescence intensity (AU, arbitrary units) of 1-kDa IRDye tracer in saline- and histone-treated mice. Mean ± S.E.M.; *n* = 3−4 mice per group. Gray box highlights coronal sections within the brain containing hippocampus region. **d** Time course of BBB permeability changes as measured by NaFl uptake in total whole brains. Mann–Whitney test; *P = 0.017. **P = 0.008; ^#^*P* = 0.031 compared to saline group, same time point; mean ± S.E.M.; *n* = 5 mice per group

To assess how circulating histones affect the paracellular permeability of the BBB, we used a histone injection mouse model in which a single non-lethal dose of histone mixture is administrated intravenously. We studied brain uptake of NaFl in mice at 24 h after saline or histone injection. In histone-treated mice, we found a regional variation of BBB permeability where histones significantly increased the uptake of NaFl in the hippocampus compared to cerebral cortex (Fig. 3a).

Next, we studied the size selectivity of the BBB opening induced by histones using different molecular size tracers. The uptake of NaFl and 1-kDa cadaverine showed higher total brain accumulation in histone-treated mice compared with the control group, further suggesting that histones make the brain vasculature leaky (Fig. 3b, left; Supplemental Fig. 3a). However, 3-kDa dextran did not show any signs of extravasation into the brain parenchyma (Fig. 3b, right; Supplemental Fig. 3b,c), indicating that histones mediate a size-selective diffusion of solutes across the paracellular pathway of the BBB.

In a separate set of experiments, we injected 1-kDa IRDye and quantified its accumulation in the brain using ex vivo NIR fluorescence imaging. Tracer distribution within the brain was analyzed in serial coronal sections at the level of + 3 mm anterior and − 5 mm posterior to the bregma. Representation of tracer accumulation on each coronal section versus distance from bregma showed the progression of the leakage as distribution of the tracer within the brain from caudal to rostral (Fig. 3c). Brain water content was not different between groups (77 ± 0.2% and 76 ± 0.1%, saline- and histone-injected mice, respectively; *n* = 6 each). Collectively, these results indicate that circulating extracellular histones increase the paracellular permeability of the BBB in a size- and region-selective manner.

To test the duration of BBB disruption (to NaFl), four groups of mice were used at 1, 3, 7, and 14 days after saline or histone injection. The uptake of NaFl increased significantly on days 1 through 7 after starting histone injection (Fig. 3d). Importantly, the peak time of BBB disruption was reached at 3 days, whereas the reversibility of the BBB opening was observed on day 14 after histone injection (Fig. 3d).

### Histones decrease expression of TJ and AJ proteins in the hippocampus

**Fig. 4:**
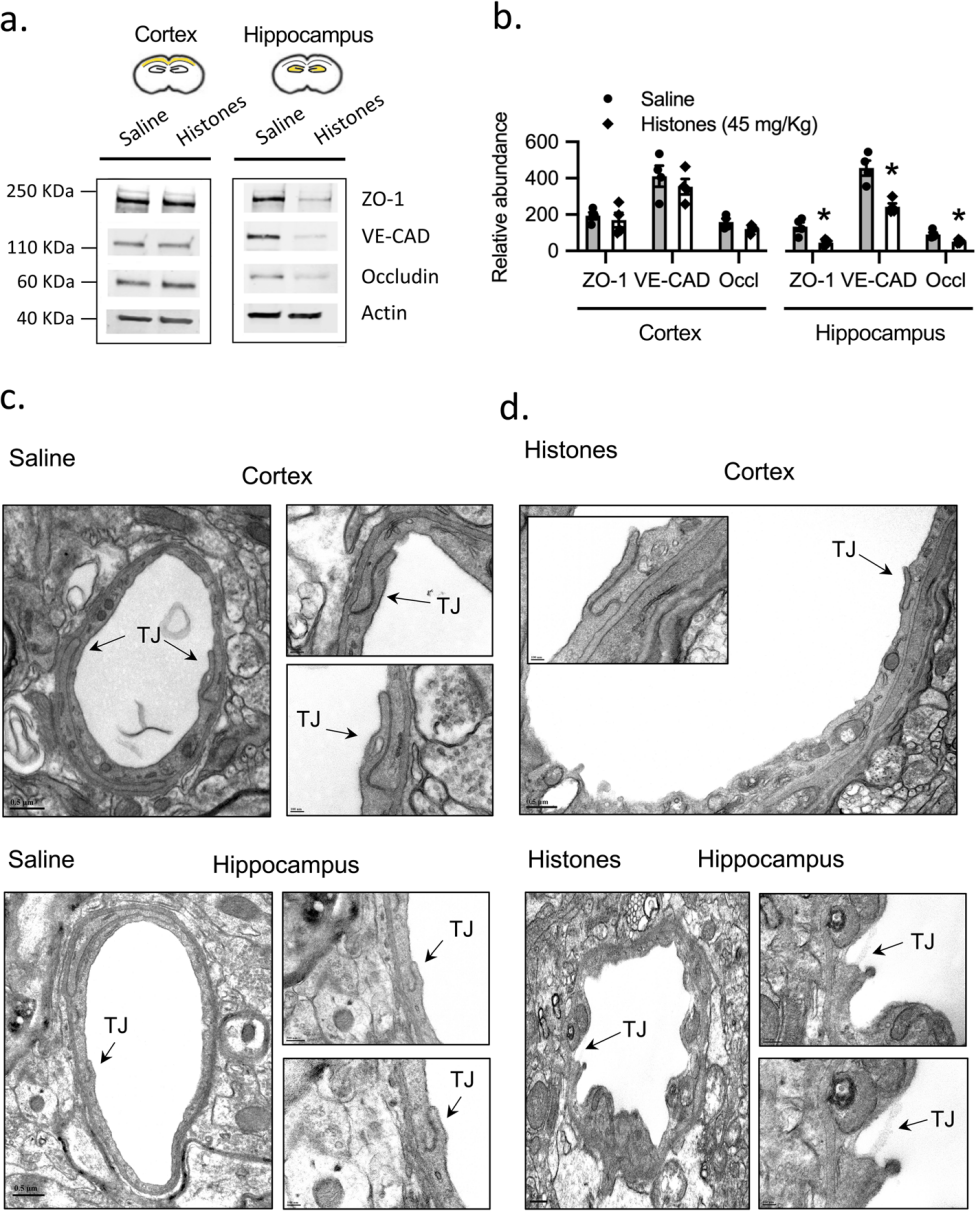
Extracellular histones reduce selectively TJ and AJ expression in the hippocampus. **a** Western blots of ZO-1, occludin, and VE-cadherin in cerebral cortex and hippocampus homogenates at 24 h post-saline or histone treatment. **b** Quantification of their protein abundance relative to ß-actin loading control. Mann–Whitney; *P < 0.05; mean ± S.E.M.; *n* = 4 mice per group. For gel source data, see Supplementary Fig. 4. **c**, **d** Electron microscopy micrographs showing capillaries in cerebral cortex and hippocampus from saline- and histones-treated mice at 24 h post-injection showing endothelial cells lining the capillary lumen and joined by TJs (arrowheads). Scale bar = 0.5 μm; inserts (100 nm, 200 nm)

The BBB is characterized by tightly sealed endothelial cells that restrict the paracellular transport of molecules into the brain parenchyma through the expression of TJs and AJs [18]. Next, we investigated whether histone-induced BBB disruption correlates with a decreased expression of TJ proteins (occludin, ZO-1) and an AJ protein (VE-cadherin). As shown in Fig. 4a, b, protein expression levels of ZO-1, occludin, and VE-cadherin in the hippocampus were reduced by 65%, 42%, and 46%, respectively, in the histone-treated group at 24 h post-treatment compared with the saline group (all *P* < 0.01). However, there was no significant attenuation of the protein levels of ZO-1, occludin, and VE-cadherin in the histone group in the cerebral cortex (Fig. 4. For gel source data, see Supplementary Fig. 4).

Furthermore, ultrastructural changes in TJ integrity at the endothelial cell-cell contact regions was investigated by electron microscopy. As shown in Fig. 4c, the cell–cell junctions between endothelial cells remained intact in the saline group and appeared as sealing intercellular clefts in brain capillaries from both cerebral cortex and hippocampus. The basal membrane was also continuous and integrated. However, as shown in Fig. 4d, circulating histones affected TJ structure in the hippocampus, while no obvious effect of histones on the structure of TJs was observed in the cerebral cortex.

**Fig. 5:**
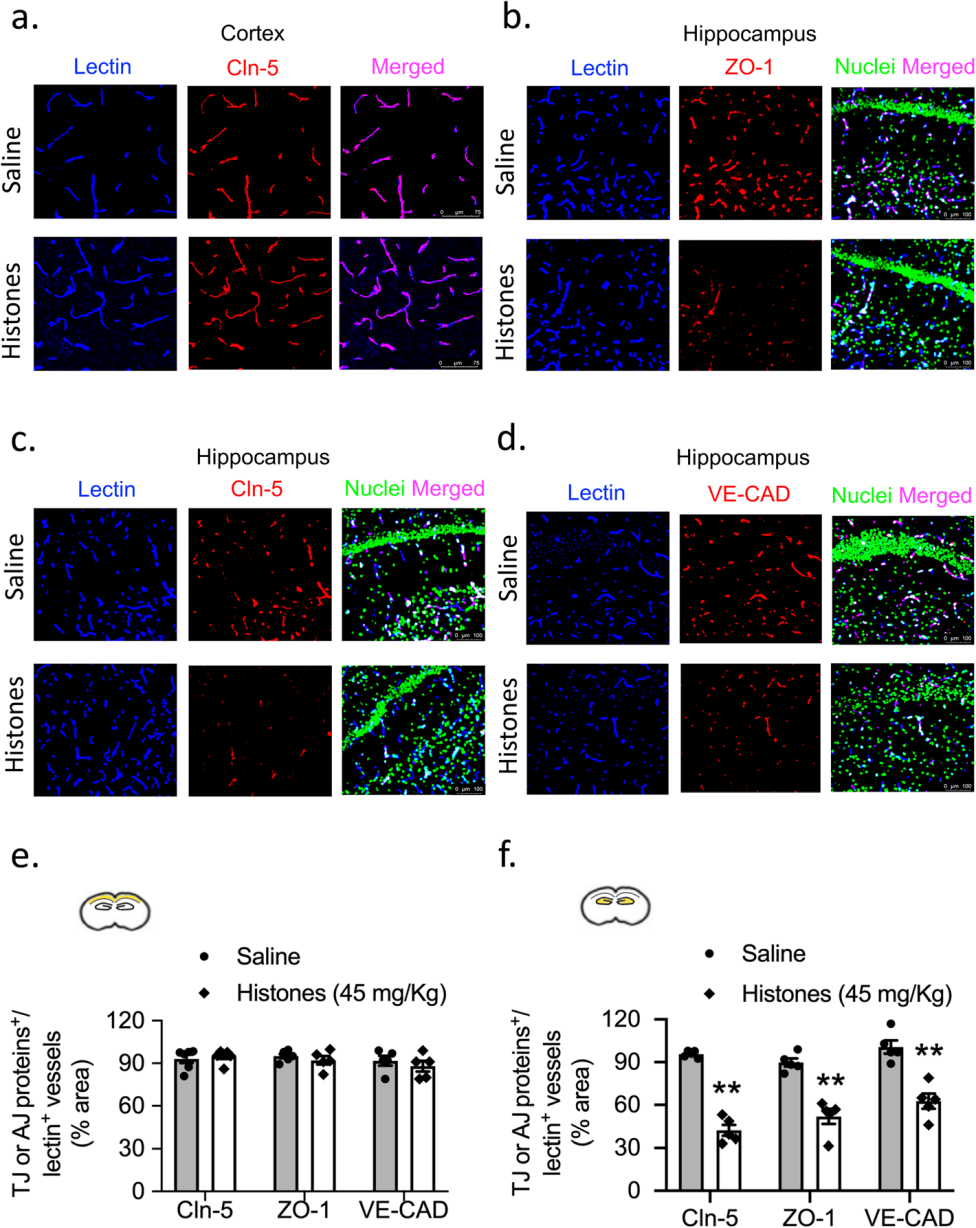
Histones decrease TJ and AJ protein expression levels in vivo. Confocal microscopy images of brain sections from **a** cerebral cortex of claudin-5 (red) co-stained with lectin (blue) and **b**–**d** from hippocampus of TJ proteins ZO-1, claudin-5, and AJ protein VE-cadherin (red) co-stained with lectin (blue). Nuclei staining (DAPI) was pseudocolored in green in hippocampus slides. **e**, **f** Quantification of ZO-1, claudin-5, and VE-cadherin coverage on lectin-positive microvessels in capillaries of cortex and hippocampus at 24 h post-saline or histone treatment. Scale bars = 75 μm (cerebral cortex), 100 μm (hippocampus). Mann–Whitney; **P < 0.01; mean ± S.E.M.; *n* = 5 mice per group

In addition, the immunostaining of TJ proteins (claudin-5 and ZO-1) and the AJ protein (VE-cadherin) was also assessed. Consistent with BBB breakdown, unlike the cerebral cortex, the microvessels in the hippocampus from histones-treated mice showed a reduction in length of claudin-5 and ZO-1, and VE-cadherin of 58%, 43%, and 37.5%, respectively, compared to those from saline-treated mice (Fig. 5).

### Circulating histones do not activate astrocytes or microglia in vivo

**Fig. 6:**
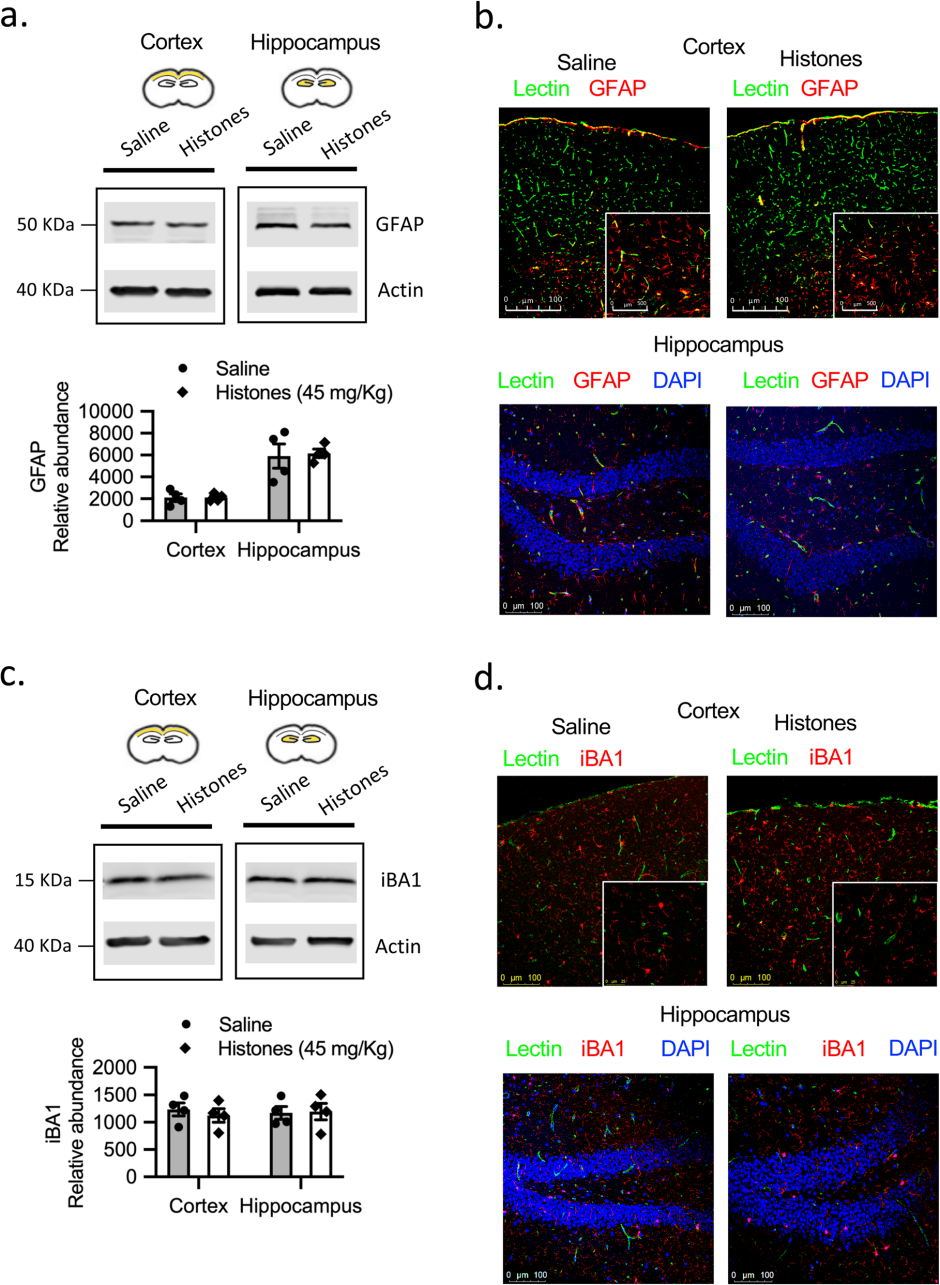
Circulating histones do not activate astrocytes or microglia in vivo. **a** Western blots of GFAP in cerebral cortex and hippocampus homogenates at 24 h post-saline or histones treatment. Quantification of GFAP protein abundance relative to ß-actin loading control. Mean ± S.E.M.; *n* = 4 mice per group. For gel source data, see Supplementary Fig. 5. **b** Confocal microscopy images of GFAP (red) and lectin (green) in cerebral cortex and hippocampus. Representative images of 3–4 mice per group. Scale bar = 100 μm. **c** Western blots of iBA1 in cerebral cortex and hippocampus homogenates at 24 h post- treatment post-saline or histones treatment. Quantification of iBA1 protein abundance relative to ß-actin loading control. Mean ± S.E.M.; *n* = 4 mice per group. For gel source data, see Supplementary Fig. 6. **d** Confocal microscopy images of iBA1 (red) and lectin (green) in cerebral cortex and hippocampus. Representative images of 3–4 mice per group. Scale bar = 100 μm

Astrocyte and microglia activation may occur concomitantly in response to direct insults inflicted to the brain or during systemic inflammation through a range of toxic circulating molecules that can access the brain microenvironment through a leaky BBB [34, 35]. The next series of experiments aimed to better understand whether histone-induced BBB disruption can result in activation of astrocytes and/or microglia in vivo. We used Western blotting to quantify the protein expression of a molecular marker of astrocytes (GFAP) and microglia (iBA1) in both cerebral cortex and hippocampus. We found no difference in levels of GFAP or iBA1 expression between groups (Fig. 6a, c. For gel source data, see Supplementary Figs. 5 and 6). As expected, we observed that GFAP staining was more conspicuous in the white matter than in the gray matter while iBA1 was uniformly distributed. Moreover, immunofluorescence staining showed no differences in GFAP- or iBA1-positive astrocytes or microglia, respectively at 1 day after histone exposure in either the hippocampus or cerebral cortex (Fig. 6b, d. For gel source data, see Supplementary Figs. 5 and 6). Together, these results indicate that histone-induced BBB dysfunction is not dependent on astrocytes or microglia.

### Changes in membrane charges determine histone-induced barrier integrity in vitro

**Fig. 7:**
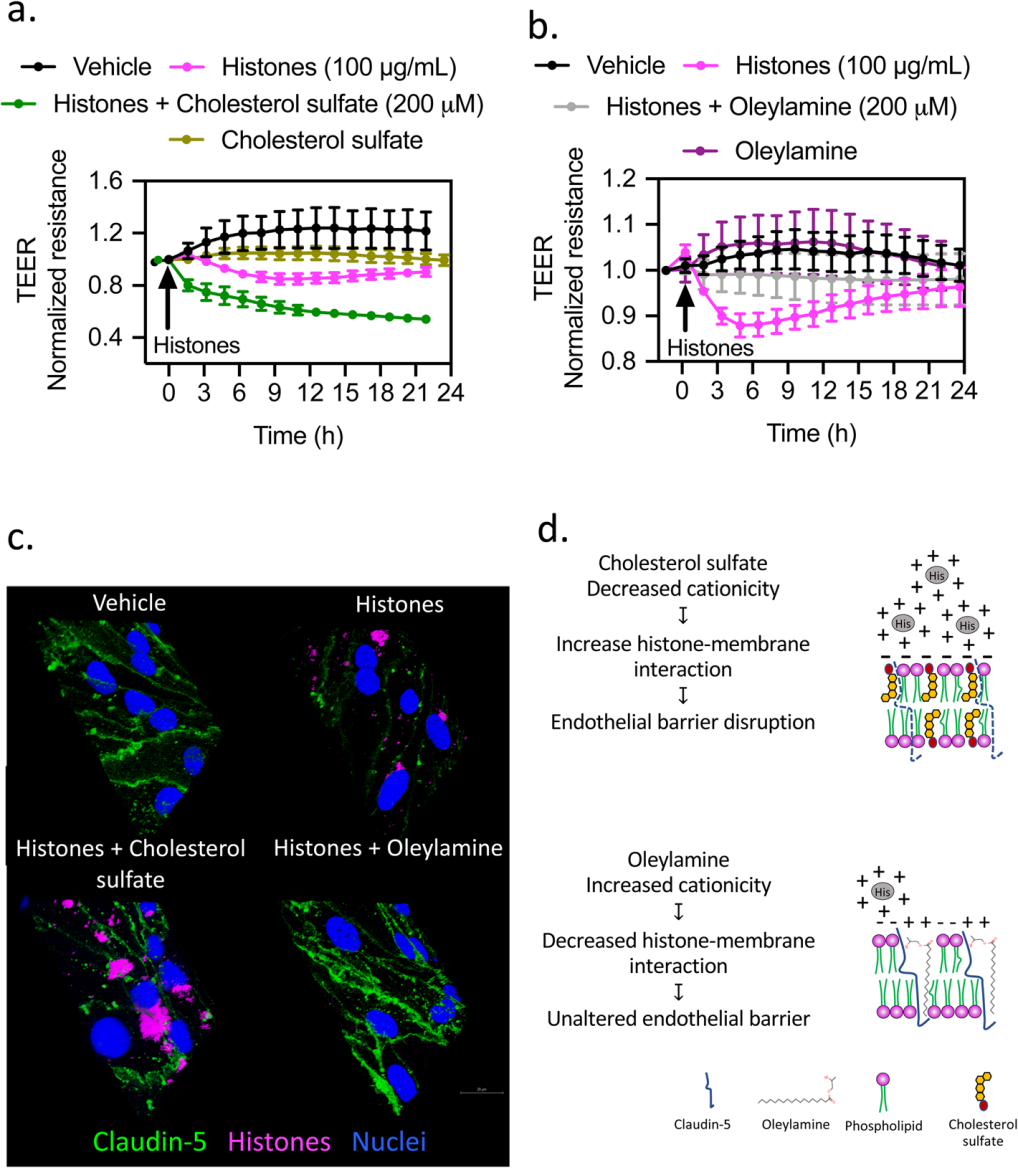
Membrane surface charge affects histone-induce changes in endothelial permeability. TEER measurements across confluent monolayers of brain endothelial cells treated with vehicle (saline) or histones (100 μg/mL) in the presence of **a** cholesterol sulfate (200 μM) and **b** oleylamine (200 μM). Mean ± S.E.M. **c** Confocal microscopy images of brain endothelial cells showing claudin-5 immunostaining after vehicle or fluorescently labeled histone exposure in the presence of cholesterol sulfate and oleylamine (200 μM). Scale bar = 20 μm. **d** Cartoon summarizing how changes in membrane cationicity affect histone-induced barrier disruption in brain endothelial cells

Based on previous reports and given the anionic nature of the plasma membrane, positive charges on histone tails may facilitate histone anchorage to cell membranes [5, 36]. Next, we tested the effect of exogenous manipulation of membrane cationicity on barrier function integrity [31]. We investigated the effect of cholesterol sulfate and oleylamine to either decrease or increase, respectively, membrane cationicity on histone-induced barrier disruption. Cholesterol sulfate enhanced histone-mediated decrease in TEER suggesting an increased histone-membrane interaction. The addition of positive charges to the plasma membrane with oleylamine inhibited histone-mediated decrease in TEER (Fig. 7b, c). Barrier integrity measurements were further confirmed with claudin-5 immunostaining. In the presence of cholesterol sulfate, histone-treated cells showed decreased claudin-5 immunostaining and increased histone molecules attached to cell membranes. Oleylamine did not alter claudin-5 immunostaining and showed a lack of histone-membrane interaction (Fig. 7c). These results indicate that histone-induced barrier disruption is due to a histone-membrane interaction that can be modulated by changes in membrane surface charges.

## Discussion

In this study, we report novel findings about the role of circulating extracellular histones in neuroinflammation by altering BBB permeability. First, our data indicate that histones disrupt TJ integrity and increase endothelial paracellular permeability in mouse brain endothelial cells accompanied with significant intercellular gap formation. The histone-induced barrier dysfunction is reversible and not caused by endothelial cell death as evidenced by negative PI staining. Furthermore, we examined the effects of histones on BBB structure and function in vivo, which to the best of our knowledge is the first study in this field. We demonstrated that systemic administration of histones in mice leads to BBB hyperpermeability mainly in the hippocampus due to a breach in endothelial cell-cell junctions. Lastly, our in vivo data also indicate that histone-injected mice exhibit a size-selective and reversible BBB opening without causing significant activation of astrocytes and microglia.

The integrity of the endothelial structure determines the barrier and normal function of the vasculature. Extensive research efforts have assessed the degree and time-dependency of tissue/organ damage in response to exogenous histone injection. Because circulating histone-induced tissue damage initiates with their direct interaction with vascular endothelial cells, the unique nature of microvascular endothelial cells within each organ/tissue becomes a determinant factor in organ susceptibility to histone damage. For instance, extracellular histone-induced lung injury has been extensively characterized [5, 37, 38]. The strong anionic charge that pulmonary endothelial cells exhibit plays a dual function. On the one hand, it maintains the hydrodynamic balance and prevents fluid leakage; on the other hand, it confers an excellent targeting site for positively charged histones [37]. In contrast, in the kidney, the fenestrated glomerular endothelium allows small and/or positively charged proteins such as histones to easily pass from the blood circulation into the Bowman space where they are subsequently excreted in the urine as early as 1 h after histone injection [38]. Thus, the susceptibility of individual organs to histone toxicity is likely varied. To date, no studies have elucidated whether systemic histones affect cerebral microvascular permeability.

We first explored the effect of histones on brain microvascular endothelial cells. Results from our in vitro data showed that histones were able to increase endothelial permeability to small molecules at an effective concentration of 100 μg/mL. The barrier dysfunction response was concomitant with decreased staining of TJ proteins, especially claudin-5 and ZO-1. We used calf thymus histones given the evidence that isolated histones obtained from critically ill patients show toxicity profiles similar to that of calf thymus histones when incubated with various human endothelial cells or injected into mice [5]. Of translational relevance is the strong positive correlation between circulating histone levels in trauma patients and soluble thrombomodulin (sTM), a marker of endothelial injury [5]. To our surprise, however, we found that histones did not affect brain endothelial cell viability even at concentrations up to 250 μg/mL. These findings differ from previous studies in cultured cells where histone exposure at > 20 μg/mL caused endothelial cell toxicity [5, 38]. Likewise, endothelial cell apoptosis has been proposed as a mechanism responsible for enhanced endothelial permeability [39]. Since we found that the general caspase inhibitor ZVAD-fmk did not prevent the loss of barrier integrity and that there was low PI uptake in histone-treated cells, we suggest that neither an induction of apoptosis through a caspase-dependent pathway nor cell death plays a fundamental role in histone-induced barrier dysfunction in brain endothelial cells. In addition to the direct effect of histones on brain endothelial cells, we were able to address the minimal impact of histone-induced endothelial cytotoxicity in vivo. In this instance, the endothelium-dependent vasodilatory response in intact cerebral arteries isolated from histone-treated mice was unaltered, compared to saline-treated mice. In stark contrast, histones at 10 μg/mL impaired endothelial-dependent vasodilation in mesenteric arteries and ultimately caused death of ~ 25% of endothelial cells [26]. Together, these observations raise the possibility that brain endothelial cells may have a protective mechanism against high levels of circulating histones in the bloodstream compared to other cell types and/or vascular beds [4, 5, 30].

The concentration range of histones used in the present study was based on the findings in healthy individuals (2.3 μg/mL) and patients with severe thoracic trauma 4 h after injury (10 to 230 μg/mL) [5]. In mouse experiments, we elected intravenous injection of an unfractionated mixture of all five histones, mimicking histone release in vivo as observed in severe inflammatory states with uncontrolled cell injury [5]. However, it is worth clarifying that in most of the existing literature, there is no clear distinction between reported levels of circulating histone proteins and nucleosomes. Thus, it is difficult to separate the pathological consequences induced by circulating free-DNA histones (“naked” histones) from the effects that are attributable to their complex in the form of extracellular nucleosomes, imposing a great challenge to data interpretation (reviewed by [40]).

To investigate whether histones cause BBB leakage in vivo, we assessed the paracellular permeability of the BBB using NaFl (376 Da), a small molecule tracer that has been extensively used to detect subtle alterations in TJ permeability [41]. In our mouse experiments, increased NaFl uptake was detected at 24 h after histone challenge, suggesting BBB leakage. Importantly, we found a regional susceptibility to histones, indicated by a significantly higher tissue accumulation of NaFl in the hippocampus compared to cerebral cortex. Consistently, we observed lower expression of TJs and AJs and opening (widening) of intercellular space in the hippocampus of histone-treated mice. Furthermore, tracer experiments revealed that the histone-induced BBB opening is limited to small molecules, but not to larger molecules. We found the molecular mass threshold to be < 3-kDa; this molecular mass cut-off indicates that most serum proteins would not extravasate. The finding of a similar brain water content in histone- and saline-treated groups further supports a minimal impact on fluid filtration. A limitation of our experiments, as well as BBB research in general based on the use of fluorescent tracers of different molecular sizes, is that these tracers differ in chemical structure and charge (lipophilicity), and thus may not be comparable to each other or reflecting in vivo pathophysiology (reviewed by [41]). The BBB permeability response in our model of histone injection appears to be subtle and size-selective, compared to animal models of Alzheimer’s disease and ischemia, where large molecules like albumin-conjugated Evan’s blue (~ 68 kDa) and IgG (~ 150 kDa) can permeate across the BBB [42–44]. However, while disruption of BBB can be shown in pathological conditions such as stroke and multiple sclerosis, showing a disrupted BBB in Alzheimer’s disease has been more controversial [45]. Indeed, some, but not all, animal models of Alzheimer’s disease exhibit increased BBB permeability [46]. It remains to be seen whether BBB hyperpermeability can be tied to neuroinflammation and whether it is the cause or consequence of multiple neurodegenerative diseases.

We also characterized BBB opening and recovery time course after histone injection. Here, we report acute opening of the BBB after histone exposure, as demonstrated by NaF extravasation, that persisted for at least 7 days with recovery of barrier integrity by 14 days. Future work is warranted to determine if cytokine release as a part of the systemic histone-induced inflammatory burst drive the delayed increase in BBB permeability and initiate secondary injury mechanisms in the brain.

As an integral part of neuroinflammation, BBB injury has been considered an important consequence of inflammatory cell activation. We thought to determine whether histone-induced BBB leakage is indirectly caused by their effect to promote a proinflammatory phenotype in astrocytes or microglia. However, our results do not support this hypothesis as histones did not alter the expression level of astrocytes and/or microglia. Of interest, our data show that BBB disruption is not necessarily linked to increased neuroinflammation. These results indicate that BBB damage is not a predictor to a neuroinflammatory response, therefore providing a compelling example of how neuroinflammation is not the mechanism of BBB disruption. However, factors that may favor neuroinflammation such as the degree of systemic inflammation and temporal profile of BBB breakdown should also be considered. Meanwhile, it is difficult to discuss whether histones are harmful to other cell types in the brain, since we (or others) have not yet explored their effects on neurons or other components of the neurovascular unit.

Consistent with the important role of BBB in maintaining CNS homeostasis, BBB disruption has been implicated in a variety of neurological disorders [47, 48]. In this regard, the linker histone H1, not extracellular core histones, has been found to cause neuronal cell death and promote microglial survival. Low concentrations of H1 upregulate major histocompatibility complex (MHC) class II receptor expression in microglia and show powerful microglial chemoattractant properties [49]. Individual histones exert different biological roles, for example, histones H3 and H4 are hallmark mediators of endothelial cell death [4, 12] while histone H4 mainly acts as an activator of platelet aggregation [12]. However, no study thus far has investigated their underlying mechanisms using in vivo systems.

Histones undergo post-translational modifications via methylation, phosphorylation, or acetylation [50]. In addition, arginine residues on histones H3 and H4 may undergo deimination by peptidyl deiminase 4 (PAD4) in neutrophils, converting arginine to citrulline which facilitates chromatin decondensation during NET formation [51]. Moreover, citrullinated histones are found in the extracellular space as the most abundant protein component of NETs [52, 53]. Previous studies from our laboratory demonstrated that recombinant citrullinated histone 3 (CitH3) caused plasma leakage in the splanchnic microcirculation and promoted endothelial AJ opening via contractile cytoskeleton reorganization [54]. Although the specific importance of citrullinated histones, relative to other modified forms of histones, is beyond the scope of the present study, further exploration of their role in regulating BBB would greatly enhance our understanding of brain inflammation.

The mechanisms underlying histones’ action are poorly understood. Several pathways have been proposed related to their cytotoxic properties, ability to disrupt plasma membranes, and direct signaling activities. A recent study also identified the lytic effect of histone H4 on smooth muscle cells and the role of NETs on fibrous cap erosion in atherosclerotic lesions [31]. A common perspective is that extracellular histones act as danger-associated molecular pattern (DAMP) proteins that interact with TLRs and activate downstream signaling. In line with this, several studies have demonstrated that histones bind to TLRs on various cells triggering sterile inflammation and platelet aggregation [10, 55, 56]. However, some of the histone actions, such as histone-induced endothelial cell calcium spike or overload and associated cytotoxicity, cannot be attributed to TLR-mediated signaling, as demonstrated in the native endothelium of *en face* mouse mesenteric arteries [5, 26]. Apparently, endothelial responses to histones are not homogeneous, consistent with literature results describing endothelial cell heterogeneity [23, 57]. Whether this is due to heterogeneous expression of a still unknown “histone receptor” or some cell-to-cell variations remains to be explored. Other intracellular pathways have also been associated with histones, such as NFΚB [9], mitogen-activated protein kinases [56], and NLRP3/caspase-1 activation [9, 13, 58–60]. Interestingly, there is an alternative theory pointing to the physical property of histones, as they are positively charged molecules that actively interact with negatively charged phospholipids in the cell membrane, a mechanism first described in 1994 by Pereira et al. [5, 31, 36] and supported by others [5, 31, 36]. Our data further support a histone-membrane interaction, since manipulation of the endothelial membrane surface charge with oleylamine (positively charged) or cholesterol sulfate (negatively charged) was able to alter histone binding and effects causing TJ disruption.

## Conclusions

The findings presented here strongly suggest a pathophysiological role of circulating histones in regulating BBB structure and function. We demonstrated for the first time that circulating histones cause brain microvascular hyperpermeability, in a size-selective (molecules < 3-kDa) and site-specific (hippocampus) manner, by disrupting endothelial cell-cell junctions.

## Supplementary information

**Additional file 1: Supplemental Figure 1.** Real time measurements of electrical resistance to monitor the formation of the endothelial barrier in mouse brain endothelial cells. Formation of the endothelial barrier assessed by measuring TEER across primary brain endothelial cell monolayers. TEER values increased over time (“growth phase”) until they reached a maximum and plateau, indicating the formation of a tight endothelial cell monolayer by day 5 post-seeding (“tight junction barrier” phase). Arrows indicate media changes since TEER measurements were performed in real time for ~10 days.

**Additional file 2: Supplemental Figure 2.** Paracellular kinetics of NaFl and 1-kDa on mouse brain endothelial cells. Accumulation (μg/cm^2^) of (a) sodium fluorescein and (b) 1-kDa dye over time showing lag time, time required for dye to accumulate in receiver compartment, and steady state. Lag time and flux of sodium fluorescein was ~1.8 min and 0.095 μg/s x cm^2^, respectively. Lag time and flux of 1-kDa dye was ~28 min and 0.048 μg/s x cm^2^, respectively.

**Additional file 3: Supplemental Figure 3.** Paracellular permeability of 1-kDa and 3-kDa tracers into the brain parenchyma. Representative NIR fluorescence images of dorsal and ventral whole-brains from saline- and histone-injected mice intravenously injected with (a) 1-kDa and (b) 3-kDa tracers. Dotted boxes represent the images showed on representative images in the main figures. (c) Lungs from 3-kDa injected animals were also imaged to show the lack of penetration of the 3-kDa dye into the brain and rule out an ineffective intravenous dye administration. (d) Summary data showing different molecular size tracer accumulation in whole brains from saline- and histone-treated animals. *n* = 4 mice per group.

**Additional file 4: Supplemental Figure 4.** Full scans of all Western blots used for quantification in main figures. Western blots of VE-cadherin, ZO-1 and occludin in cerebral cortex and hippocampus homogenates at 24 h post-saline or histones treatment. Dotted boxes indicate lanes presented as representative blots in the respective main figures. *n* = 4 mice per group.

**Additional file 5: Supplemental Figure 5.** Full scans of all Western blots used for quantification in main figures. Western blots of GFAP in cerebral cortex and hippocampus homogenates at 24 h post-saline or histones treatment. Dotted boxes indicate lanes presented as representative blots in the respective main figures. *n* = 4 mice per group.

**Additional file 6: Supplemental Figure 6.** Full scans of all Western blots used for quantification in main figures. Western blots of iBA1 in cerebral cortex and hippocampus homogenates at 3-days post-saline or histones treatment. Dotted boxes indicate lanes presented as representative blots in the respective main figures. *n* = 4 mice per group.

## Data Availability

The data that support the findings of this study are available from the corresponding author upon reasonable request.

## Abbreviations

AJs: Adherens junctions
BBB: Blood-brain barrier
CNS: Central nervous system
ECIS: Electric cell-substrate impedance sensor
HUVECs: Human umbilical vein endothelial cells
NaF: Sodium fluorescein
NIR: Near infrared
NETs: Neutrophil extracellular traps
PBS: Phosphate buffer saline
PI: Propidium iodide
SEM: Standard error of the mean
sTM: Soluble trombomodulin
TJs: Tight junctions
TLR: Toll-like receptor
TEER: Transendothelial electrical resistance
TEM: Transmission electron microscopy
ZO-1: Zona-occludens-1
