# Supplementary figures and images for: Site-specific opening of the blood-brain barrier by extracellular histones
Source: J Neuroinflammation. 2020 Sep 22;17:281. doi: 10.1186/s12974-020-01950-x (PMC7510151; doi:10.1186/s12974-020-01950-x)

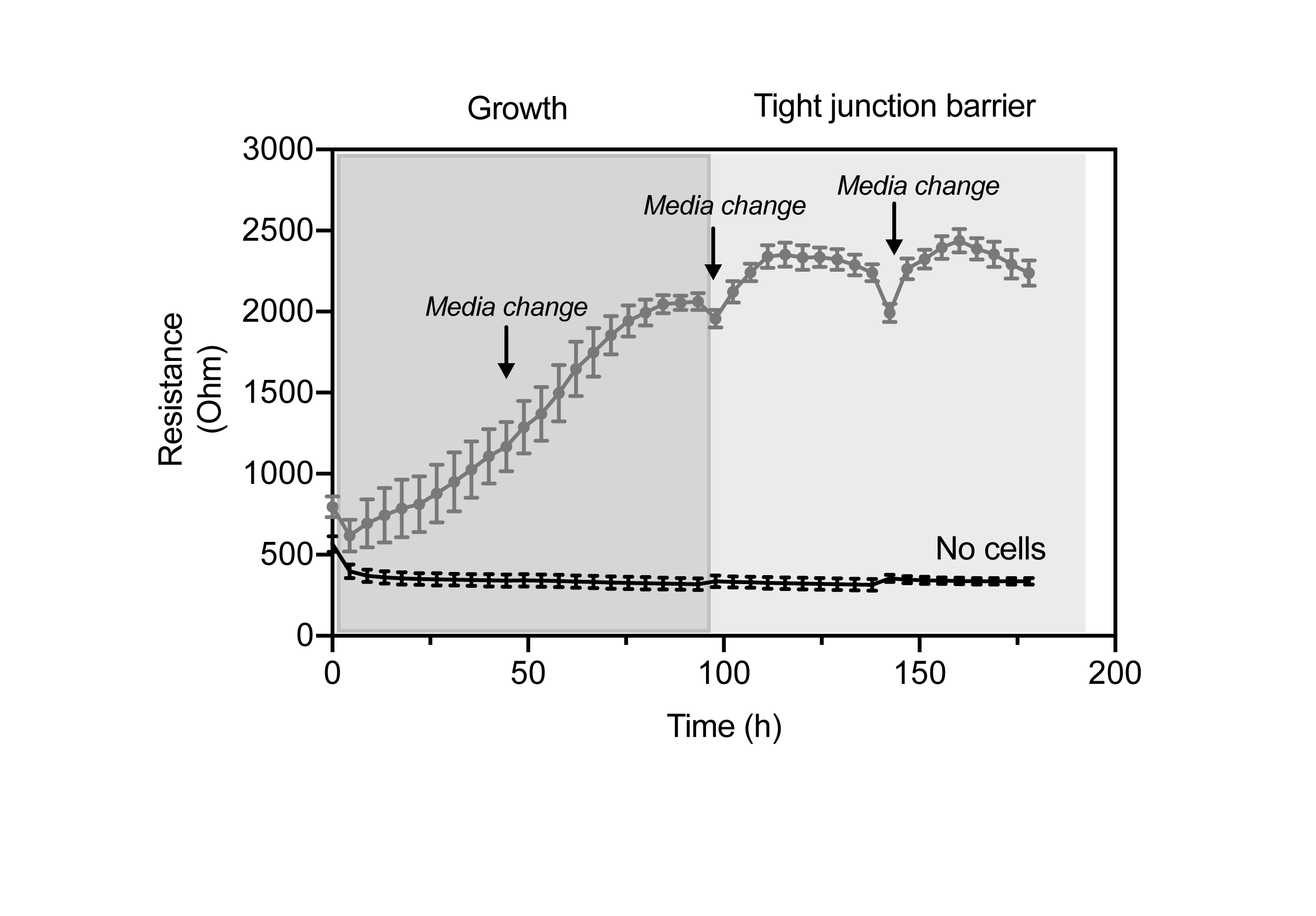

Supplement: Supplementary file 1 — Additional file 1: Supplemental Figure 1. Real time measurements of electrical resistance to monitor the formation of the endothelial barrier in mouse brain endothelial cells. Formation of the endothelial barrier assessed by measuring TEER across primary brain endothelial cell monolayers. TEER values increased over time (“growth phase”) until they reached a maximum and plateau, indicating the formation of a tight endothelial cell monolayer by day 5 post-seeding (“tight junction barrier” phase). Arrows indicate media changes since TEER measurements were performed in real time for ~10 days. [file 12974_2020_1950_MOESM1_ESM.tiff]

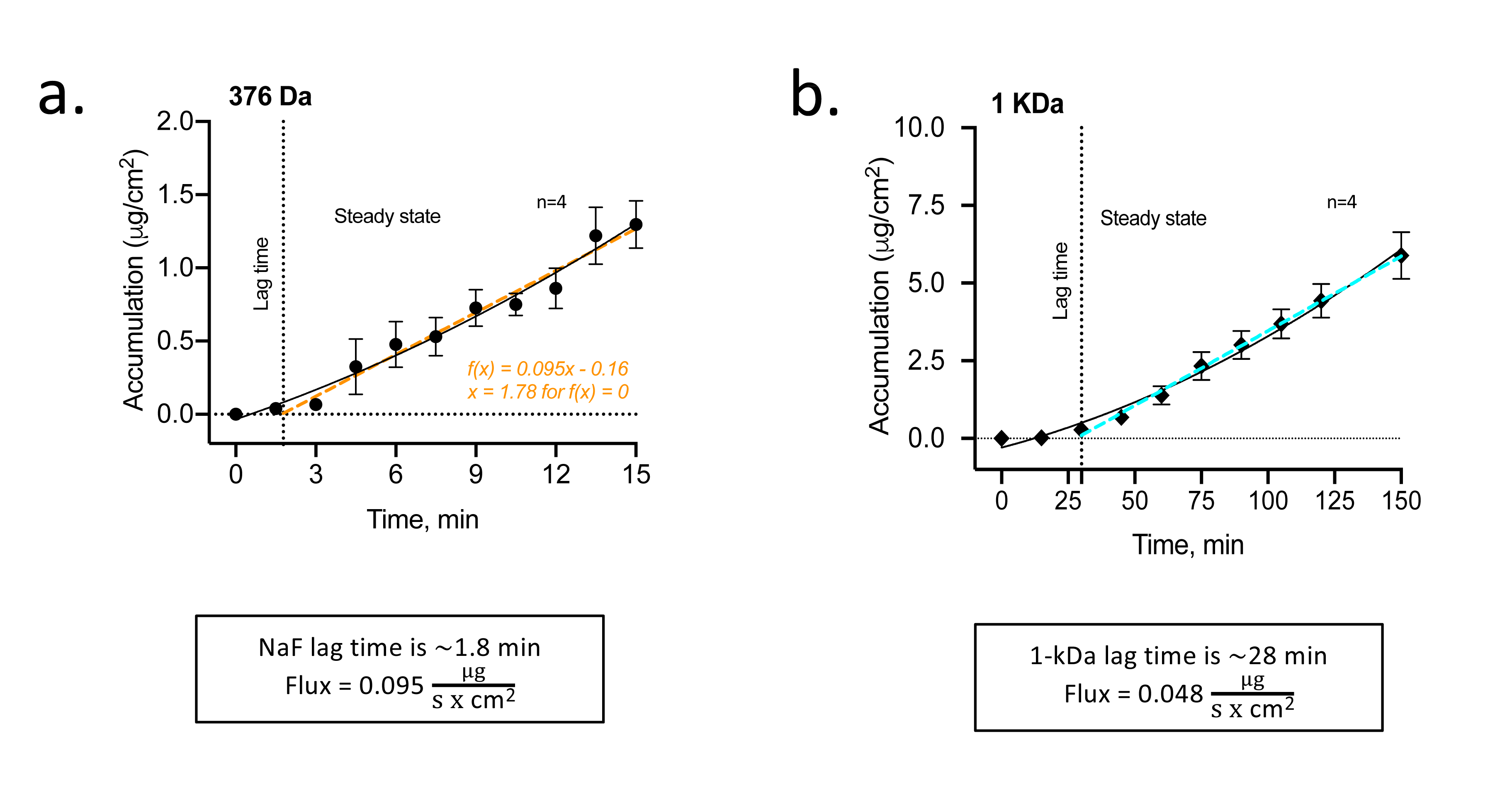

Supplement: Supplementary file 2 — Additional file 2: Supplemental Figure 2. Paracellular kinetics of NaFl and 1-kDa on mouse brain endothelial cells. Accumulation (μg/cm2) of (a) sodium fluorescein and (b) 1-kDa dye over time showing lag time, time required for dye to accumulate in receiver compartment, and steady state. Lag time and flux of sodium fluorescein was ~1.8 min and 0.095 μg/s x cm2, respectively. Lag time and flux of 1-kDa dye was ~28 min and 0.048 μg/s x cm2, respectively. [file 12974_2020_1950_MOESM2_ESM.tiff]

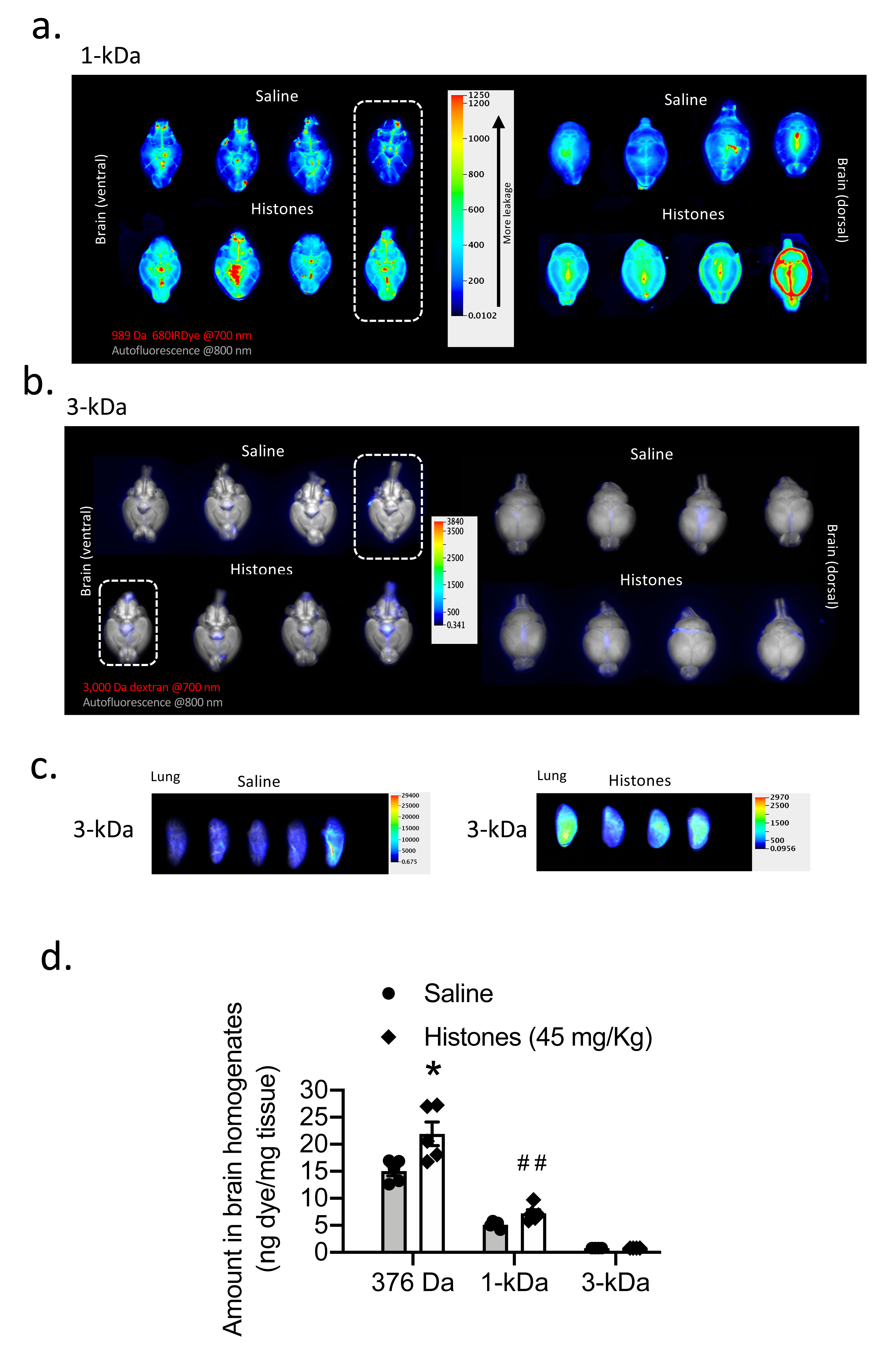

Supplement: Supplementary file 3 — Additional file 3: Supplemental Figure 3. Paracellular permeability of 1-kDa and 3-kDa tracers into the brain parenchyma. Representative NIR fluorescence images of dorsal and ventral whole-brains from saline- and histone-injected mice intravenously injected with (a) 1-kDa and (b) 3-kDa tracers. Dotted boxes represent the images showed on representative images in the main figures. (c) Lungs from 3-kDa injected animals were also imaged to show the lack of penetration of the 3-kDa dye into the brain and rule out an ineffective intravenous dye administration. (d) Summary data showing different molecular size tracer accumulation in whole brains from saline- and histone-treated animals. n = 4 mice per group. [file 12974_2020_1950_MOESM3_ESM.tiff]

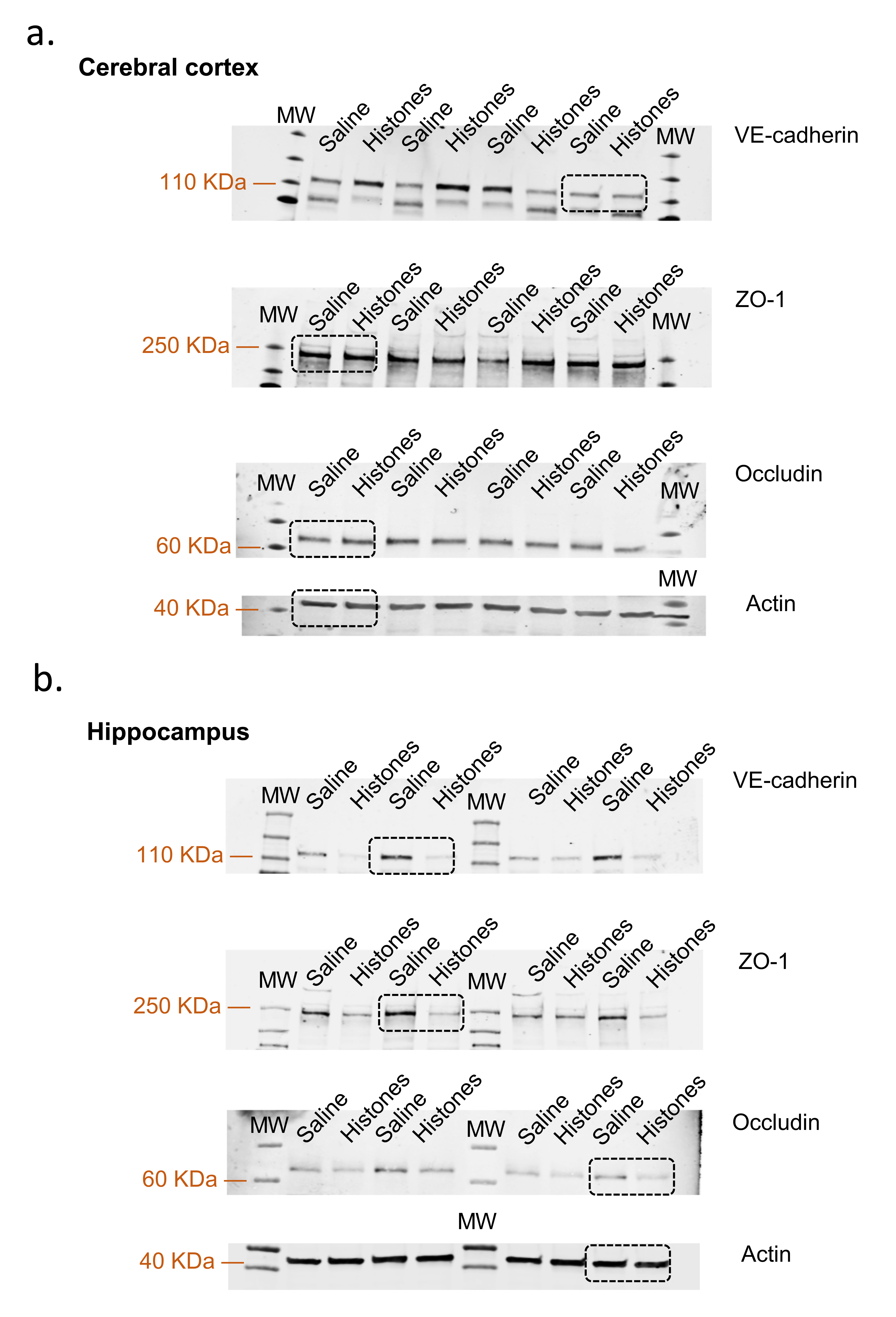

Supplement: Supplementary file 4 — Additional file 4: Supplemental Figure 4. Full scans of all Western blots used for quantification in main figures. Western blots of VE-cadherin, ZO-1 and occludin in cerebral cortex and hippocampus homogenates at 24 h post-saline or histones treatment. Dotted boxes indicate lanes presented as representative blots in the respective main figures. n = 4 mice per group. [file 12974_2020_1950_MOESM4_ESM.tiff]

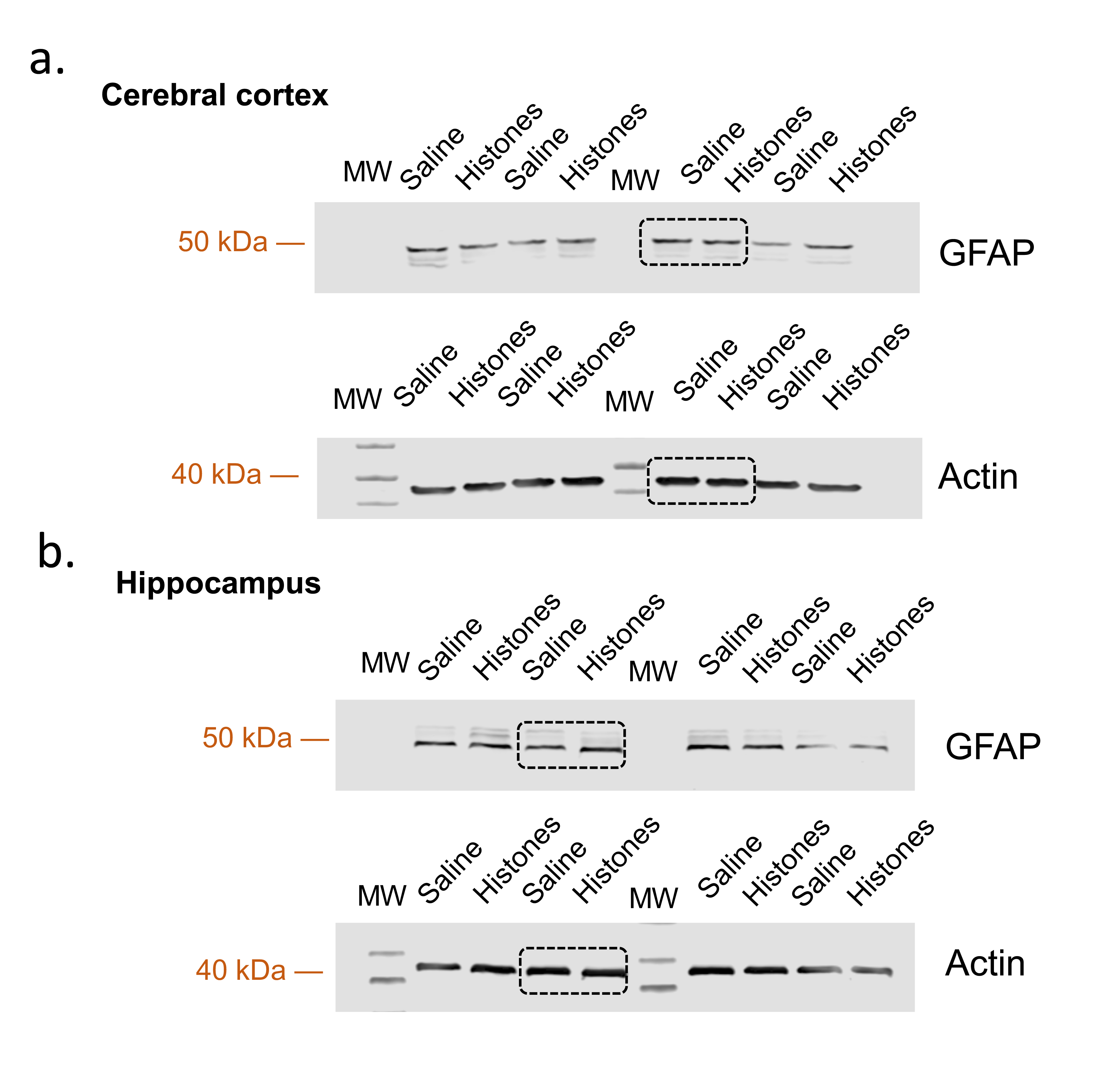

Supplement: Supplementary file 5 — Additional file 5: Supplemental Figure 5. Full scans of all Western blots used for quantification in main figures. Western blots of GFAP in cerebral cortex and hippocampus homogenates at 24 h post-saline or histones treatment. Dotted boxes indicate lanes presented as representative blots in the respective main figures. n = 4 mice per group. [file 12974_2020_1950_MOESM5_ESM.tiff]

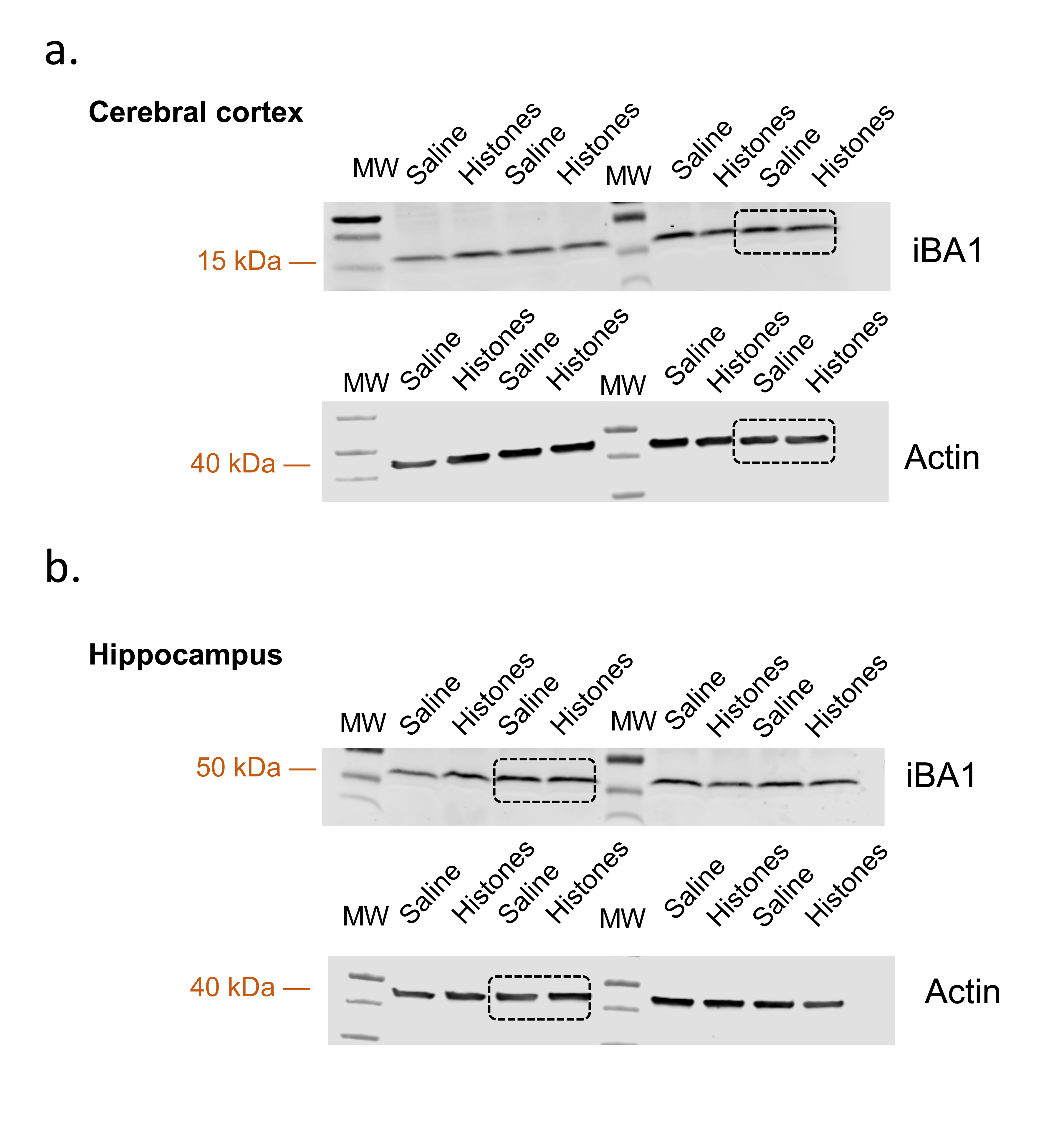

Supplement: Supplementary file 6 — Additional file 6: Supplemental Figure 6. Full scans of all Western blots used for quantification in main figures. Western blots of iBA1 in cerebral cortex and hippocampus homogenates at 3-days post-saline or histones treatment. Dotted boxes indicate lanes presented as representative blots in the respective main figures. n = 4 mice per group. [file 12974_2020_1950_MOESM6_ESM.tiff]
